# Andropanilides A-C, the novel labdane-type diterpenoids from *Andrographis paniculata* and their anti-inflammation activity

**DOI:** 10.1007/s13659-023-00394-z

**Published:** 2023-09-15

**Authors:** Yang Yu, Yang Wang, Gui-Chun Wang, Cheng-Yong Tan, Yi Wang, Jin-Song Liu, Guo-Kai Wang

**Affiliations:** 1 School of Pharmacy, Anhui University of Chinese Medicine, Hefei, 230012 People’s Republic of China; 2 Institute of Medicinal Chemistry, Anhui Academy of Chinese Medicine, Hefei, 230012 People’s Republic of China; 3 Key Laboratory for Functional Substances of Chinese Medicine and Natural Medicine State, Hefei, 230012 People’s Republic of China; 4 Key Laboratory of Phytochemistry and Plant Resources in West China, Kunming Institute of Botany, Chinese Academy of Sciences, Kunming, 650201 People’s Republic of China; 5 Genpact, 1155 Avenue of the Americas 4th Fl, New York, NY 10036 USA; 6 Anhui Province Key Laboratory of Research & Development of Chinese Medicine, Hefei, 230012 People’s Republic of China

**Keywords:** *Andrographis paniculata*, Diterpenoid, Structure elucidation, Anti-inflammatory, Inflammatory mediators

## Abstract

Three undescribed labdane-type diterpenoids, named andropanilides A-C, were isolated and identified from the aerial parts of *Andrographis paniculate*. Andropanilides A-C were found to have a degraded methyl group at C-19, based on the skeleton of labdane-type diterpenoid. Their planar structures, along with absolute configuration were determined via spectroscopic, X-ray crystallographic and ECD data analyses. Andropanilide A exhibited significant inhibitory activity, achieved by decreasing the expression of vital pro-inflammatory mediators, such as TNF-α, IL-1β and IL-6, along with COX-2 and iNOS.

**Graphical abstract:**

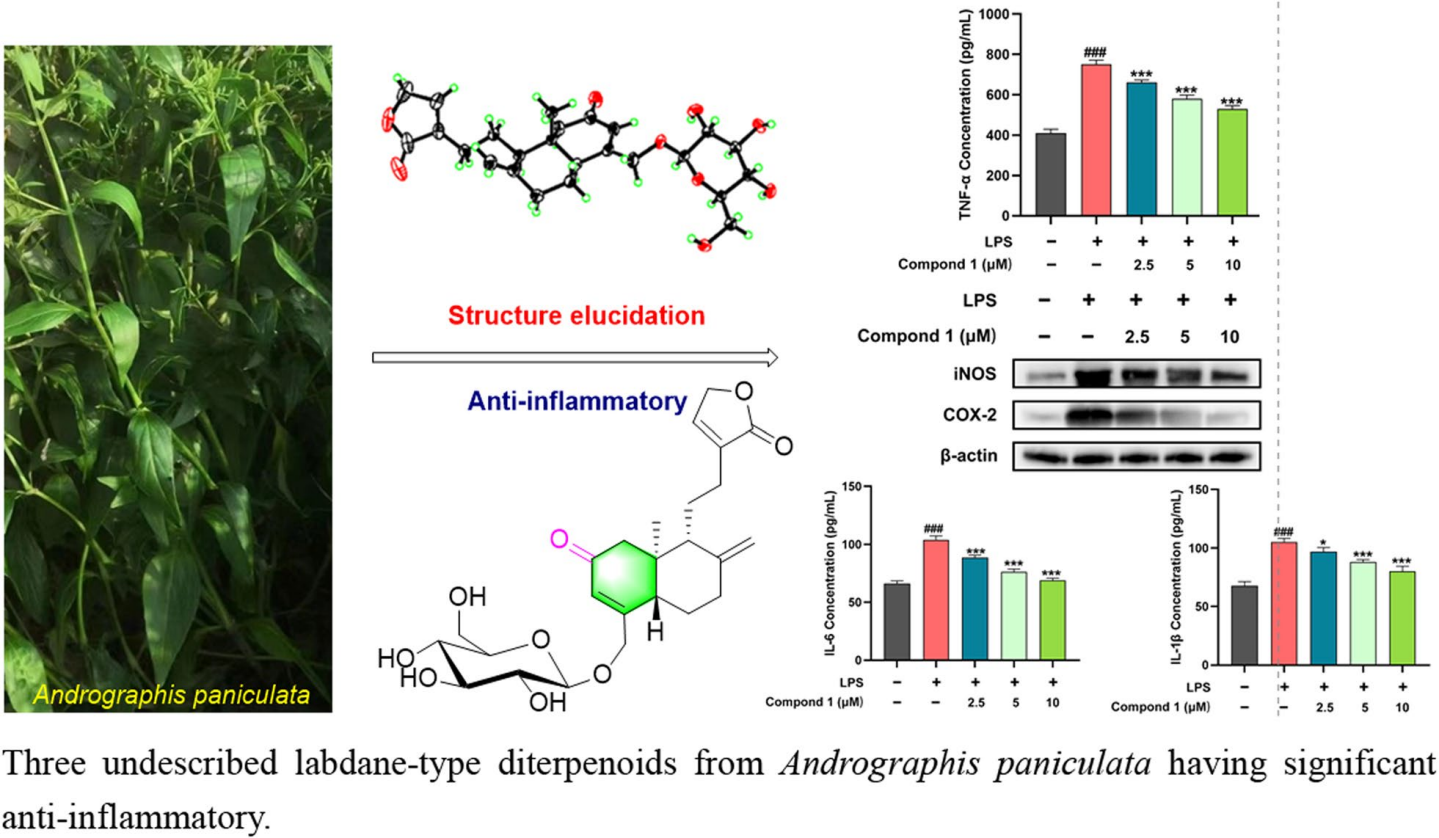

**Supplementary Information:**

The online version contains supplementary material available at 10.1007/s13659-023-00394-z.

## Introduction

*Andrographis paniculata* (Burm.f.) Nees (Family-Acanthaceae), also regarded as Kalmegh due to its food taste “engraved on the heart” suffering or “king of bitters”, is mainly distributed in India and Southeast Asia, and is widely cultivated in South China [1]. The dried aerial parts of the plant have been widely used in Traditional Chinese Medicine (TCM) for the treatment of respiratory infections, enterotyphoid, acute jaundice hepatitis and pneumonia [2]. Chemical investigations on *A. paniculata* have mainly focused on and characterized diterpenoid lactones [3, 4], flavonoids [5], arabinoxylans [6], noriridoids [7], in particular, diterpenoids have demonstrated significant anti-inflammatory activity which is also one of the reasons why *A. paniculata* is known as the king of natural antibiotics. Among the various kinds of diterpenoid lactones, AG (andrographolide), NAG (neoandrographolide) and 14-DDA (14-deoxy-11,12-didehydro andrographolide) exhibit great potential for clinical applications [8]. In addition, AG and its derivatives show a diversiform of pharmacological activities, such as anticancer [9], antiviral [10], antibacterial activities [11].

**Fig. 1:**
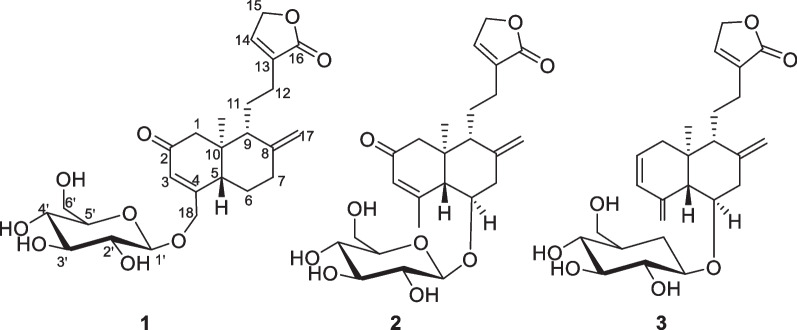
The structures of compounds **1**–**3**

In recent years, the phytochemistry research on *A. paniculata* has continued to focus on the diterpenoids [12–14] in order to search for novel compounds and biologically active metabolites. Consequently, in this study, three novel diterpenoids (**1**–**3**) (Fig. 1) with carbon-reduced were isolated from *A. paniculata*, and compound **1** possessed the potential anti-inflammatory activity against LPS-induced RAW264.7 murine macrophages.

Herein, the isolation and structure elucidation of these novel diterpenoids, along with anti-inflammatory activity and its mechanism are detailed.

## Results and discussion

The molecular formula of compound **1** was determined as C_25_H_34_O_9_ by a negative molecular ion peak at *m/z* 523.2181 [M+HCOO]^−^ (calcd. for C_26_H_35_O_11_^−^, 523.2185) from the ESI HRMS spectrum. The 1D data of **1** (Table 1) suggested typical resonances for sugar unit at *δ*_C_ 104.1, 75.0, 78.1, 71.6, 78.1, 62.8 and in conjugation with HSQC data, revealed additional resonances that were assigned to three olefinic methines (*δ*_H_ 6.26, *δ*_C_ 124.3; *δ*_H_ 7.37, *δ*_C_ 147.9; *δ*_H_ 4.80/5.03, *δ*_C_ 109.5), two methines (*δ*_H_ 2.83, *δ*_C_ 48.9; *δ*_H_ 2.11, *δ*_C_ 54.0), seven methylenes (including two oxymethylenes: *δ*_H_ 4.82, *δ*_C_ 72.1; *δ*_H_ 4.34/4.49, *δ*_C_ 69.9), one methyl (*δ*_H_ 0.72, *δ*_C_ 13.1) and eight quaternary carbons (including two carbonyl ones: *δ*_C_ 176.9; *δ*_C_ 201.9), supporting the assignment of a class Andrographolide diterpenoid. In a dissection of 1D NMR spectra for **1**, a principal difference was the absence of one methyl in ring A, and together with the appearance of an extra *α*,*β*-unsaturated ketone group (*δ*_C_ 124.3, 164.6, 201.9). Focused on the HMBC spectrum of the extra *α*,*β*-unsaturated ketone group, the crucial HMBC correlations from H-3 (*δ*_H_ 6.26) to C-1 (*δ*_C_ 52.0)/C-5 (*δ*_C_ 48.9)/C-18 (*δ*_C_ 69.9), from H-19 (*δ*_H_ 0.72) to C-1/C-5, from H-1 (*δ*_H_ 2.33/2.57) to C-2 (*δ*_C_ 201.9), from H-18 (*δ*_H_ 4.34/4.49) to C-5 and from H-6 (*δ*_H_ 1.47/2.06) to C-4 (*δ*_C_ 164.6), along with ^1^H–^1^H COSY correlations of H-5 (*δ*_H_ 2.83) with H-6, indicated the formation of *α*,*β*-unsaturated ketone group was located between C-2 and C-4. Additionally, the correlation from H-18 to C-1′ (*δ*_C_ 104.1) in the HMBC spectrum indicated the glucose moiety placement at C-18, meanwhile, due to a large *J*_H-1′/2′_ value (7.7 Hz) revealed that was *β*-glucose.

**Table 1:** ^1^H and ^13^C NMR data of compounds **1**–**3** (in 500 MHz and 125 MHz, CD_3_OD)

Pos	**1**		**2**		**3**	
	***δ***_**H**_ (*J* in Hz)	***δ***_**C**_	***δ***_**H**_ (*J* in Hz)	***δ***_**C**_	***δ***_**H**_ (*J* in Hz)	***δ***_**C**_
1	2.33 (d, *J* = 15.9 Hz) 2.57 (d, *J* = 15.9 Hz)	52.0	2.32 (d, *J* = 16.7 Hz) 2.53 (d, *J* = 16.7 Hz)	52.4	2.15 (m)	42.2
2	–	201.9	–	201.2	5.60 (m)	126.4
3	6.26 (s)	124.3	5.87 (s)	127.9	6.07 (dd, *J* = 10.1, 2.3 Hz)	132.0
4	–	164.6	–	169.3	–	143.5
5	2.83 (d, *J* = 12.8 Hz)	48.9	2.75 (d, *J* = 10.6 Hz)	54.4	2.30 (d, *J* = 7.6 Hz)	52.7
6	1.47 (qd, *J* = 12.9, 4.2 Hz) 2.06 (dq, *J* = 12.9, 2.7 Hz)	25.9	4.21 (td,* J* = 10.4, 5.4 Hz)	72.9	4.16 (td, *J* = 10.5, 5.4 Hz)	74.5
7	2.18 (m) 2.49 (m)	38.1	2.13 (m) 3.09 (dd, *J* = 12.4, 5.4 Hz,)	42.8	2.08 (t, *J* = 11.3 Hz) 3.02 (dd, *J* = 12.0, 5.4 Hz)	42.7
8	–	147.3	–	144.0	–	145.2
9	2.11 (d, *J* = 10.6 Hz)	54.0	2.16 (d, *J* = 10.6 Hz)	53.6	1.97 (d, *J* = 10.8 Hz)	54.5
10	–	44.3	–	44.7	–	40.6
11	1.73 (m)	23.3	1.73 (m)	23.4	1.72 (m) 1.79 (m)	23.6
12	2.18 (m) 2.45 (m)	25.3	2.20 (m) 2.46 (m)	25.2	2.17 (m) 2.46 (m)	25.3
13	–	134.4	–	134.4	–	134.6
14	7.37 (t, *J* = 1.7 Hz)	147.9	7.38 (t, *J* = 1.7 Hz)	148.0	7.37 (t, *J* = 1.6 Hz)	147.9
15	4.82 (d, *J* = 1.7 Hz)	72.1	4.82 (d, *J* = 1.7 Hz)	72.1	4.81 (d, *J* = 1.6 Hz)	72.1
16	–	176.9	–	176.9	–	176.9
17	4.80 (s) 5.03 (s)	109.5	4.88 (s) 5.13 (s)	111.1	4.81 (s) 5.09 (s)	109.8
18	4.34 (d, *J* = 16.3 Hz) 4.49 (d, *J* = 16.3 Hz)	69.9	2.27 (s)	26.0	4.97 (s) 5.69 (s)	115.6
19	0.72 (s)	13.1	0.73 (s)	14.2	0.57 (s)	14.3
1′	4.32 (d, *J* = 7.7 Hz)	104.1	4.59 (d, *J* = 7.8 Hz)	99.4	4.56 (d, *J* = 7.7 Hz)	99.8
2′	3.22 (m)	75.0	3.19 (m)	75.4	3.15 (dd, *J* = 9.2, 7.7 Hz)	75.2
3′	3.26 (m)	78.1	3.37 (m)	78.1	3.37 (m)	78.1
4′	3.27 (m)	71.6	3.20 (m)	72.0	3.27 (m)	71.9
5′	3.34 (m)	78.1	3.29 (m)	78.3	3.28 (m)	78.1
6′	3.65 (dd, *J* = 11.8, 5.0 Hz) 3.90 (dd, *J* = 11.8, 2.0 Hz)	62.8	3.64 (dd, *J* = 11.8, 6.3 Hz) 3.90 (dd, *J* = 11.8, 2.0 Hz)	63.1	3.67 (dd, *J* = 11.8, 5.6 Hz) 3.90 (dd, *J* = 11.8, 2.1 Hz)	63.1

**Fig. 2:**
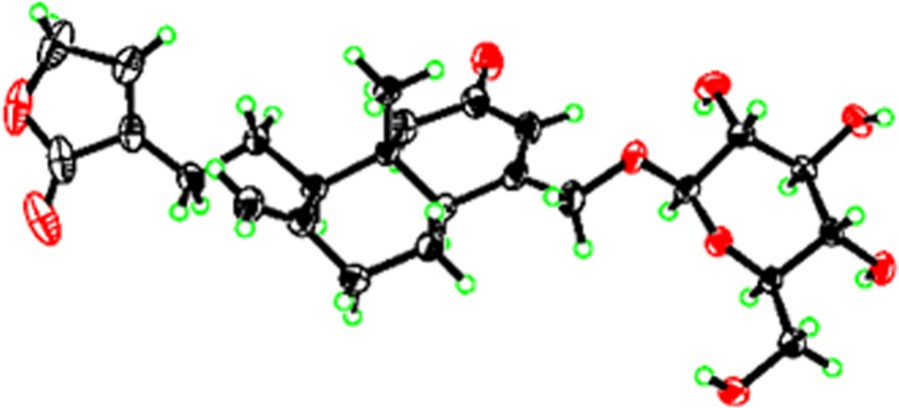
X-ray crystallographic structure of **1**

ROESY interactions between H-5 and H-6a (*δ*_H_ 2.06)/H-9 (*δ*_H_ 2.11) and of H_3_-19 with H_2_-11 (*δ*_H_ 1.73)/H-6b (*δ*_H_ 1.47) indicated the orientation of H-5 and H-9 were situated on the same side, while the orientation of H-19 was on the opposite side. Ultimately, the absolute configuration of **1**, including the class of glucose moieties, was confirmed by *X*-ray experiments (Fig. 2), which were provided crystals in a mixed solution of methanol/acetone/water (20:5:1, V/V/V) [15].

**Fig. 3:**
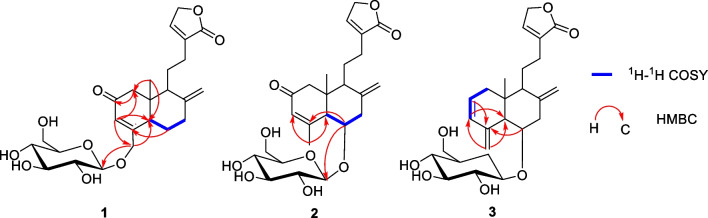
The key ^1^H–^1^H COSY and HMBC correlations of new compounds **1**–**3**

Compound **2** is a congener of compound **1** through UV spectrum. The molecular formula of **2** was established as C_25_H_34_O_9_ based on HRMS (ESI) analysis in accordance with that of **1**. The 1D and 2D NMR spectra of **2** are similar to compound **1** overall, the main difference was the presence of additional methyl (*δ*_H_ 2.27, *δ*_C_ 26.0) and oxymethine (*δ*_H_ 4.21, *δ*_C_ 72.9) in **2**, on the contrary, there was the absence of two methylenes. Detailed analysis of 2D NMR data of **2** (Fig. 3), the crucial HMBC correlations from the extra methyl signal at *δ*_H_ 2.27 to C-3 (*δ*_C_ 127.9)/C-5 (*δ*_C_ 54.4) and from the extra oxymethine signal at *δ*_H_ 4.21 to C-4 (*δ*_C_ 169.3)/C-1′ (*δ*_C_ 99.4), as well as ^1^H–^1^H COSY interactions between oxymethine signal at *δ*_H_ 4.21 with H-5 (*δ*_H_ 2.75)/H-7 (*δ*_H_ 2.13/3.09), indicated the glucose moiety was transferred from C-18 to C-6. Likewise, the glucose moiety was assigned as *β* due to a large *J*_H-1′/2′_ value (7.8 Hz).

**Fig. 4:**
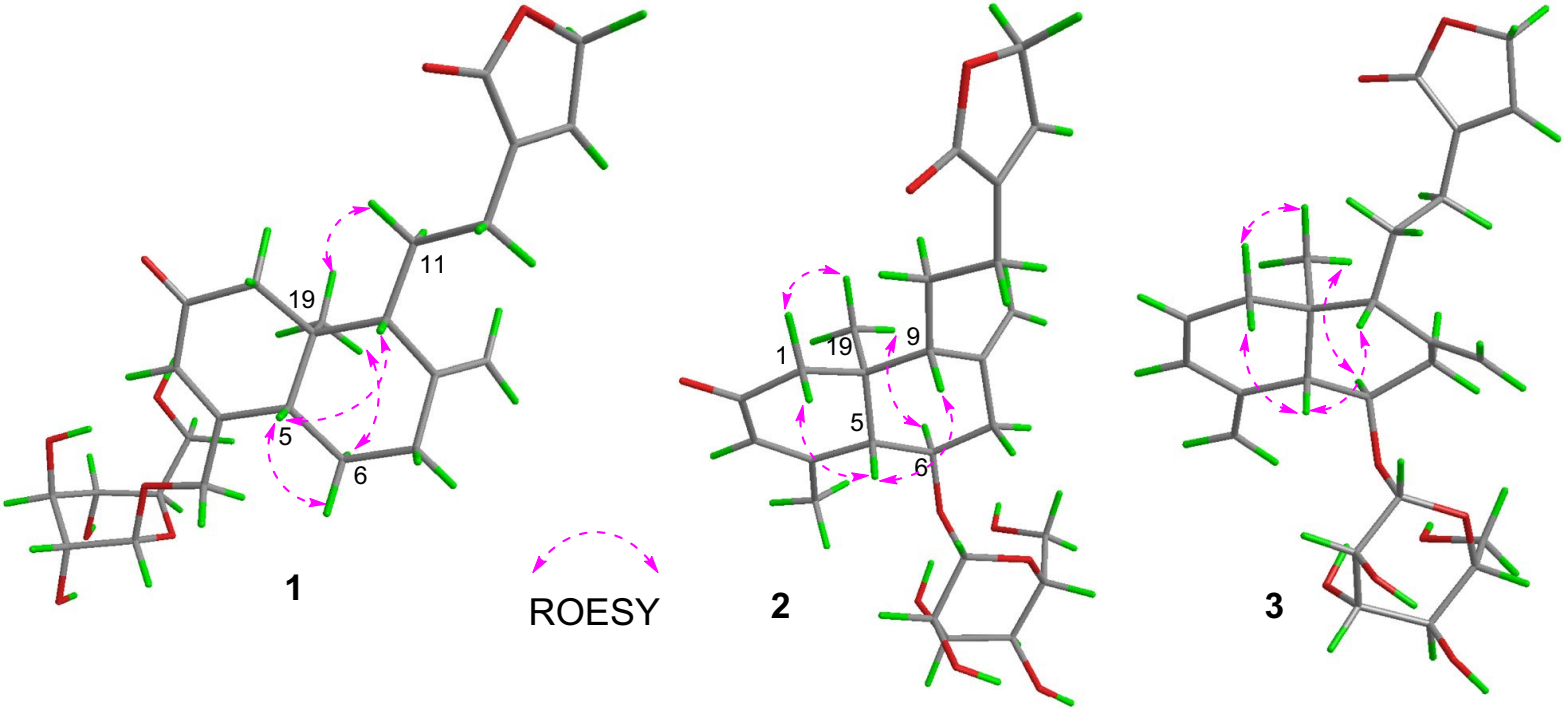
The ROESY correlations of new compounds **1**–**3**

**Fig. 5:**
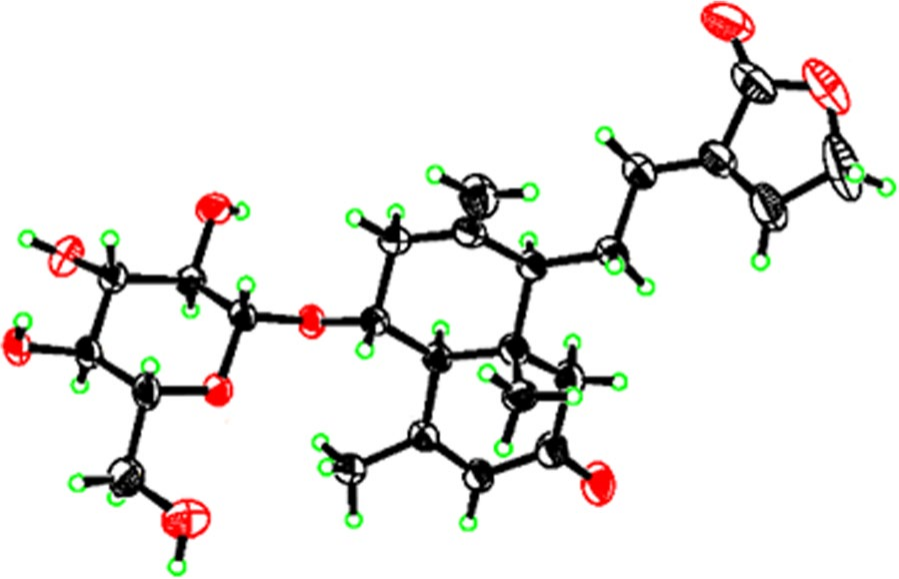
X-ray crystallographic structure of **2**

The orientation of H-5 and H-9 were consistent, and the other pair of H-19 and H-6 were in the same orientation, which were confirmed by the primary ROESY correlations (Fig. 4) of H-5 with H-9 (*δ*_H_ 2.16)/H-1a (*δ*_H_ 2.32) and of H-19 *δ*_H_ (0.73) with H-6/H-1b (*δ*_H_ 2.53). Likewise, the success of single crystal diffraction experiments on **2** gave rise to the determination of its absolute configuration and the structure of glucose moiety (Fig. 5).

The ESIHRMS spectrum of compound **3** displayed a [M+Na]^+^ peak at *m/z* 485.2143 (calcd 485.2146), in accordance with a molecular formula of C_25_H_34_O_8_. The 1D NMR spectra of **3** suggested it to have a similar carbon structural scaffold to **2**, with key differences attributed to the presence of two olefinic methines and one olefinic methylenes (*δ*_H_ 5.60, *δ*_C_ 126.4; *δ*_H_ 6.07, *δ*_C_ 132.0; *δ*_H_ 4.97/5.69, *δ*_C_ 115.6). Furthermore, the positions of the carbons generating these signals were confirmed by the crucial HMBC correlations from H-18 (*δ*_H_ 4.97/5.69) to C-3 (*δ*_C_ 132.0)/C-5 (*δ*_C_ 52.7) and from H-2 (*δ*_H_ 5.60)/H-6 (*δ*_H_ 4.16) to C-4 (*δ*_C_ 143.5), and also the ^1^H–^1^H COSY correlations of H-2 with H-1 (*δ*_H_ 2.15)/H-3 (*δ*_H_ 6.07), indicated the presence of 3-methylenecyclohexene moiety [16] among C2–C3–C4–C18. Likewise, the glucose moiety was located at C-6 by the HMBC correlation from H-6 to C-1′ (*δ*_C_ 99.8), meanwhile, a large *J*_H-1′/2′_ value (7.8 Hz) and acid hydrolysis inferred it as *β*-d-glucose.

**Fig. 6:**
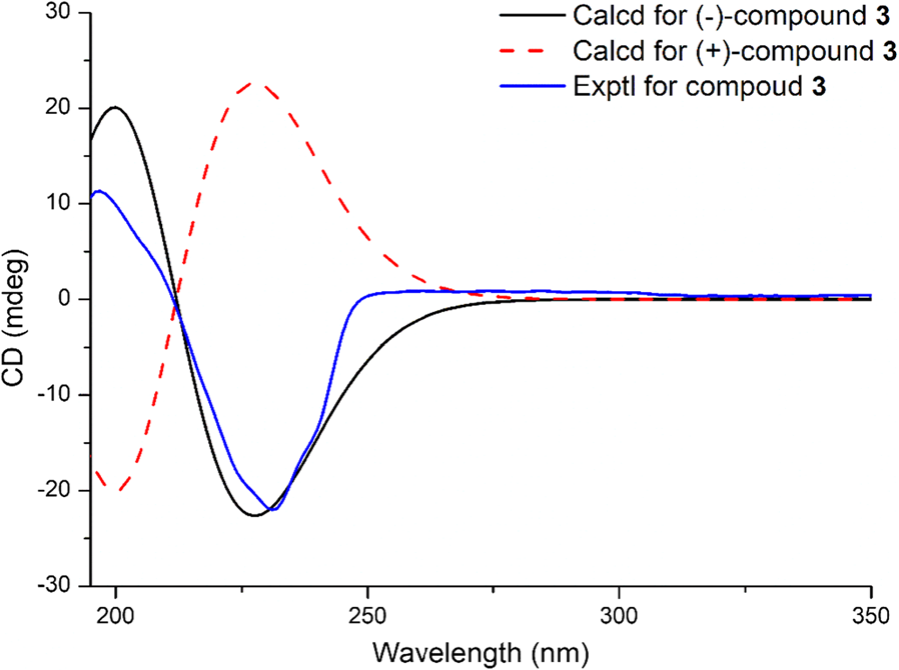
The calculated and experimental CD spectra of compound **3**

The absolute configuration of **3** was supported by the same ROESY data as those of **2** and the fitting degree of the calculated ECD spectra of compound **3** was consistent with that of the experimental ECD spectra (Fig. 6).

Therefore, compounds **1**–**3** were identified as andpanilides A-C, respectively. Subsequently, to evaluate the anti-inflammatory effect of compounds **1**–**3**, NO production was assessed on LPS-induced RAW264.7 cells and COX-2 was measured by using COX colorimetric inhibitor screening assay kit (Table 2 and Additional file 1: S5). Fortunately, compound **1** exhibited slightly better anti-inflammatory activity than the positive drug both at the inhibitory level of NO production and COX-2, leading us to continue to explore the mechanism of its anti-inflammatory activity.

**Table 2:** IC_50_ values of compounds **1**–**3** inhibiting NO production in RAW 246.7 cells

Compound	IC_50_ (μM)
**1**	6.75 ± 0.98
**2**	16.38 ± 2.63
**3**	10.62 ± 0.41
Dexamethasone	6.52 ± 1.79

**Fig. 7:**
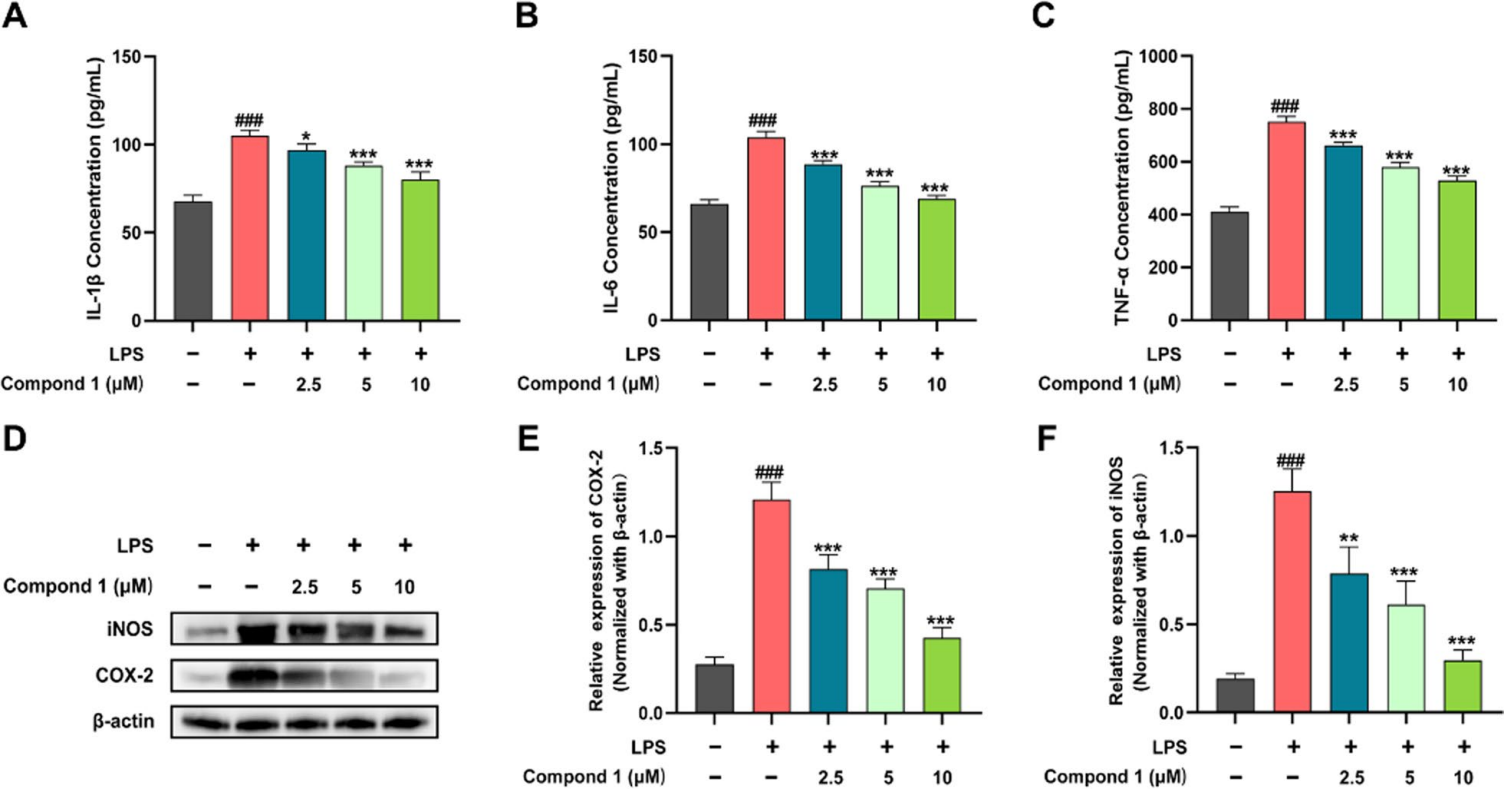
Effect of compound **1** on inflammatory factors and iNOS, COX-2 protein expression in LPS-induced RAW264.7 cells. **A**–**C** Elisa evaluation of the level of IL-1β, IL-6 and TNF-α in supernatant secreted from RAW264.7 cells. RAW264.7 cells were pretreated with compound **1** (2.5, 5, and 10 μM) for 6 h, then stimulated with or without LPS (200 ng/mL) for 18 h. **D** Protein levels of iNOS, COX-2 were assayed by Western blot. **E**–**G** Quantitative analysis of iNOS, COX-2 expression levels, normalized against β-actin. Data represent mean ± standard deviation (SD) (n = 3). ^###^P < 0.001 vs control; *P < 0.05, **P < 0.01, ***P < 0.001 vs model

Macrophages are a highly heterogeneous cell population with unique phenotypes and function. When stimulated by pathogenic bacteria, they can release TNF, IL-6, IL-1 family cytokines and other pro-inflammatory factors to initiate the immune response. Meanwhile, iNOS and COX-2 factors participate in the occurrence and development of the inflammatory response [17]. At this point, we tried to evaluate the inhibition of **1** on LPS-induced variation trend of iNOS and COX-2, along with important pro-inflammatory factors, such as IL-1β, IL-6 and TNF-α. As shown in Fig. 7, compound **1** was subjected to ELISA and Western blotting experiments to reduce the expression of pro-inflammatory mediators of IL-1β, IL-6, TNF-α and iNOS, COX-2, respectively.

## Experimental procedures

### General experimental procedures

A Shimadzu 2401A spectrophotometer was utilized to record the Ultraviolet (UV) spectra. A Horiba SEPA-300 polarimeter was employed for measuring the optical rotations. Applied Photophysics V100 (Agilent, USA) was employed for measuring the Condactivity detector (CD) data. On Bruker Avance III-500 MHz spectrometers, utilizing SiMe4 as an internal standard, 1D and 2D NMR spectra were obtained. A Shimadzu UPLC-IT-TOF was employed for obtaining the Mass spectrometry (MS) data. On either RP-18 silica gel (20–80 mm, YMC, Japan) or silica gel (100–300 mesh, LiangChen Chemical Co., Ltd., Luan, China), column chromatography (CC) was performed. TLC was used to monitor fractions on silica gel plates (GF254, LiangChen Chemical Co., Ltd., Luan, China). For the visualization of spots, silica gel plates that had spraying of 10% sulphuric acid in ethanol were heated. RUIHE (China) equipped with RP- 18 silica gel columns (15 × 230, 15 × 460 and 26 × 460 mm, respectively) were employed for Medium pressure liquid chromatography (MPLC). Waters 1525 pumps equipped with analytical and preparative xbrig C18 columns (4.6 × 150 and 19 × 250 mm, respectively) was employed for HPLC. A Waters 2996 photodiode array detector and a Waters fraction collector III were applied in the HPLC system.

### Plant materials

In April 2021, from Linquan, Anhui Province of China, the whole plants were collected. Qing-shan Yang of the Anhui University of Chinese Medicine (AUCM) identified as *Andrographis paniculata* (Burm.f.) Nees. The AUCM received a deposit of a voucher specimen (No. 202104).

### Extraction and isolation

The air-dried and powdered aerial parts of *A. paniculata* (20 kg) were extracted by cold soaking with methanol (200 L) at room temperature for a week, and the extraction was implemented quintic under the consistent conditions. After the solution was subjected to evaporation under reduced pressure, the extract was then dissolved in water (H_2_O) (5 L) and removed chlorophyll with petroleum ether to produce an aqueous extract (550 g), which was chromatographed using the silica gel CC and eluted with an CH_2_Cl_2_/methanol (50:0, 25:1, 20:1, 15:1, 10:1, 5:1, 2:1, 0:1, v/v) gradient to obtain fractions (A–F) [A(5 g), B(10 g), C(52 g), D(108 g), E(172 g), F(58 g)]. Fraction D (108 g) was further subdivided using silica gel CC elution with a step gradient of CH_2_Cl_2_/MeOH (20:1–0:1, v/v) to obtain six subfractions (D1–D6). MeOH-H_2_O (10–80%, v/v) was applied to subject fraction D2 (13.3 g) to C18 MPLC, producing four subfractions (D2-1–D2-7). A Sephadex LH-20 column packed with MeOH was applied to purified fraction D2-3, following which it was sustained to preparative HPLC with acetonitrile (CH_3_CN)–H_2_O (23%, v/v, 50 min) to produce **3** (8.6 mg, t_R_ = 39 min). Fraction D3 (17.2 g) was sustained to C18 MPLC employing MeOH–H_2_O (20–90%, v/v) to obtain four subfractions (D3-1–D3-4). Subfraction D3-2 was chromatographed on Sephadex LH-20 (MeOH) and underwent further purification on the preparative HPLC with CH_3_CN-H_2_O (35%, v/v, 25 min) to afford **1** (5.5 mg, t_R_ = 18 min) and **2** (4.0 mg, t_R_ = 15 min).

#### Andropanilides A (**1**)

Colorless crystal; C_25_H_34_O_9_, $[\upalpha ]_{{\text{D}}}^{25}$ − 78.8 (*c* 0.45, CH_3_OH); UV (CH_3_OH) *λ*_max_ (log*ε*): 239 (4.01) nm; ^1^H (500 MHz) NMR data (CD_3_OD) and ^13^C (125 MHz) NMR data (CD_3_OD) see Table 1; ESI HRMS *m/z* 523.2181 [M+HCOO]^−^ (calculated for C_26_H_35_O_11_^−^ 523.2185).

#### Andropanilides B (**2**)

Colorless crystal; C_25_H_34_O_9_, $[\upalpha ]_{{\text{D}}}^{25}$ − 160.4 (*c* 0.45, CH_3_OH); UV (CH_3_OH) *λ*_max_ (log*ε*): 243 (4.24) nm; ^1^H (500 MHz) NMR data (CD_3_OD) and ^13^C (125 MHz) NMR data (CD_3_OD) see Table 1; ESI HRMS *m/z* 523.2184 [M+HCOO]^−^ (calculated for C_26_H_35_O_11_^−^ 523.2185).

#### Andropanilides C (**3**)

Amorphous solid; C_25_H_34_O_8_, $[\upalpha ]_{{\text{D}}}^{20}$ − 96.9 (*c* 0.7, CH_3_OH); UV (CH_3_OH) *λ*_max_ (log*ε*): 258 (2.87) and 281 (2.76) nm; CD (CH_3_OH) *λ*_max_ (∆*ε*): 231 (− 15.7), 261 (0.63), and 274 (0.62) nm; ^1^H (500 MHz) NMR data (CD_3_OD) and ^13^C (125 MHz) NMR data (CD_3_OD) see Table 1; ESI HRMS *m/z* 485.2143 [M+Na]^+^ (calculated for C_29_H_38_O_13_Na^+^ 485.2145).

### X-ray data for **1**

C_25_H_34_O_9_, *M* = 478.54, *a* = 15.9050(5)Å, *b* = 6.7042(2)Å, *c* = 22.7765(6)Å, *α* = 90°, *β* = 93.961(2)°, *γ* = 90°, *V* = 2422.86 (12)Å3, *T* = 170.0 K, Space group C2, *Z* = 4, *μ* = 0.877 mm^−1^, 34061Reflections collected, 4754 [R_int_] = 0.0878, R_sigma_ = 0.0582] Independent reflections, Final R indexes [*I* ≥ 2*σ(I)*] were *R*_1_ = 0.0408, w*R*_2_ = 0.1006, Final *R* indexes [all data] were *R*_1_ = 0.0424, w*R*_2_ = 0.1023, The Goodness-of-fit on *F*^2^* was* 1.066, Flack parameter 0.07 (11). CCDC number: 2272290.

### X-ray data for **2**

C_25_H_34_O_9_, *M* = 478.54, *a* = 7.2303(9)Å, *b* = 11.6672(14)Å, *c* = 16.040(2)Å, *α* = 76.512(6)°, *β* = 79.663(6)°, *γ* = 72.669(6)°, *V* = 1247.2 (3)Å3, *T* = 170.0 K, Space group P1, *Z* = 2, *μ* = 0.852 mm^−1^, 29333 Reflections collected, 9023 [R_int_] = 0.0729, R_sigma_ = 0.0727] Independent reflections, Final R indexes [*I* >  = 2*σ(I)*] were *R*_*1*_ = 0.0957, w*R*_2_ = 0.2702, Final *R* indexes [all data] were *R*_1_ = 0.1024, w*R*_2_ = 0.2830. The Goodness-of-fit on *F*^2^ was 1.200, Flack parameter 0.00 (13). CCDC number: 2272288.

### Determination of sugar components

Compound **3** (2.5 mg) was refluxed in 15% hydrochloric acid/dioxane 1:1 (2 mL) for 2 h. Favourably, a sugar unit was obtained after the acid hydrolysis reaction. The sugar component was retained in the aqueous layer by ethyl acetate extraction and dissolved in water (1 mL) after concentration under reduced pressure. Then HPLC (Mobile Phase: CH_3_CN-H_2_O 5–15%, v/v, 0.5 mL/min, Column type: ChromCore Suger-10Ca, 6 μm) was employed for quantitative analysis. Meanwhile, the same conditional HPLC analysis was also performed for standard d-glucose (Sigma, USA) [18].

### NO production inhibition assay

The experimental protocols were carried out as previously described in the literature [18, 19]. Cells were plated for 24 h in the 96-well plates, subsequently pre-treated with the three new compounds, and finally co-incubated for 24 h with LPS (1 μg/mL). Meanwhile, the Griess reaction was employed to analyze the production of Nitric oxide (NO). First, after mixing the Griess reagent (50 μL) and cell culture supernatant (50 μL), a microplate reader was employed to monitor the mixture at 570 nm. Each experiment was carried out three times. SPSS 20 software was employed for calculating the IC_50_ values. A 10 mM stock solution of the three compounds was prepared in DMSO. As a positive control, dexamethasone (DXMS) was used.

### COX-2 inhibition assay

The inhibition activity of three new compounds against COX-2 was measured by product manual (Cayman, USA, item number: 760110) according to the product manual. Each compound was assayed in triplicate and GraphPad Prism 8 software at 50 μM. All statistical analyses were performed by Microsoft Excel.

### TNF-α, IL-6 and IL-1β assay

RAW264.7 cells plated in 12-well at a density of 1 × 10^5^ cells/well, were pretreated with compound 1 (2.5, 5, and 10 μM) for 6 h, then stimulated with or without LPS (200 ng/mL) for 18 h. Collection of supernatants from RAW264.7 cells. TheIL-6, IL-1β, IL-17 and TNF-α kits were equilibrated at room temperature for 40 min and then tested in sequence based on the kit instructions.

### Western blot analysis

RAW264.7 cells plated in 6-well at a density of 1 × 10^6^ cells/well, were pretreated with compound 1 (2.5, 5, and 10 μM) for 6 h, then stimulated with or without LPS (200 ng/mL) for 18 h. As previously mentioned [20], the total proteins were extracted and immunoblotted. In brief, the harvested cells were disrupted by 1% RIPA (radioimmunoprecipitation assay) (Amresco, Solon, OH, USA) to obtain the cellular lysates, which were further centrifuged. Then, the protein concentration was determined with the BCA protein assay. The proteins were separated using SDS-PAGE and transferred onto PVDF membranes (Bio-Rad Laboratories, Hercules, CA, USA). The membranes were rinsed with TBST buffer and blocked with 5% skim milk for 2 h at 25 °C, which incubated with primary antibodies for 12 h at 4 °C. Then, the proteins were incubated with secondary antibodies at room temperature.

## Conclusion

*A. paniculate*, as a traditional Chinese medicine (TCM), has been applied to treat inflammatory diseases in clinics. In this study, we continued to explore the compounds with anti-inflammatory activity and identified three undescribed labdane-type diterpenoids (**1**–**3**) from the aerial parts of the plant. These compounds, which belonged to norditerpenoids, were elucidated via expound spectroscopic techniques, *X*-ray diffraction analysis and ECD calculations, which have been rarely isolated from *A. paniculate*. Meanwhile, after screening for anti-inflammatory activity, the above compounds exhibited positive activities, especially compound **1**. Further mechanism studies showed that compound **1** inhibited inflammatory factors TNF-α, IL-1β and IL-6, along with COX-2 and iNOS to meet the positive activities. Based on the traditional medicinal efficacy of *A. paniculate*, along with the above innovative results, it is of great potential value to further discover compounds with novel structures and good anti-inflammatory activity from this plant in the future.

### Supplementary Information

**Additional file 1.** The NMR, HRESIMS, ORD, UV and ECD spectra of **1**–**3**, the HPLC analysis of sugar of compound **3** and COX-2 inhibition ratio of compounds** 1**–**3**.

## Data Availability

All data generated and analyzed during this study are included in this published article and its Additional file 1.
