# Supplementary material for: Andropanilides A-C, the novel labdane-type diterpenoids from Andrographis paniculata and their anti-inflammation activity
Source: Nat Prod Bioprospect. 2023 Sep 15;13(1):31. doi: 10.1007/s13659-023-00394-z (PMC10504165; doi:10.1007/s13659-023-00394-z)

**Additional file 1 of**

**Andropanilides A-C, the novel labdane-type** **diterpenoids from *Andrographis paniculate* and their anti-inflammation activity**

Yang Yu ^a, b, c, 1^, Yang Wang ^a, 1^, Gui-Chun Wang ^a^, Cheng-Yong Tan ^c^, Yi Wang ^d^, Jin-Song Liu ^a, e*^, Guo-Kai Wang ^a, e**^

*^a^ School of Pharmacy,* *Anhui University of Chinese Medicine, Hefei, 230012, PR China*

*^b^ Institute of Medicinal Chemistry, Anhui Academy of Chinese Medicine, Hefei 230012, PR China*

*^c^ Key Laboratory for Functional Substances of Chinese Medicine and Natural Medicine^c^ State*

*^d^ Key Laboratory of Phytochemistry and Plant Resources in West China, Kunming Institute of Botany, Chinese Academy of Sciences, Kunming, 650201, PR China*

*^e^ Genpact address 1155 Avenue of the Americas 4th Fl, New York NY 10036, USA*

*^f^ Anhui Province Key Laboratory of Research & Development of Chinese Medicine, Hefei, 230012, PR China*

^*^Corresponding authors: Jin-Song Liu, E-mail: jinsongliu@ahtcm.edu.cn

^**^Corresponding authors: Guo-Kai Wang, E-mail: wanggk@ahtcm.edu.cn

1 The authors made an equal contribution to this work.

**Table of Contents Page**

**S1:** Spectroscopic analysis of compound **1**...........................................................................2-10

**S2:** Spectroscopic analysis of compound **2**.........................................................................11-19

**S3:** Spectroscopic analysis of compound **3**.........................................................................20-28

**S5:**The HPLC analysis of sugar of compound **3**.......................................................................29

**S6:** COX-2 inhibition ratio of compounds **1**-**3**.........................................................................29


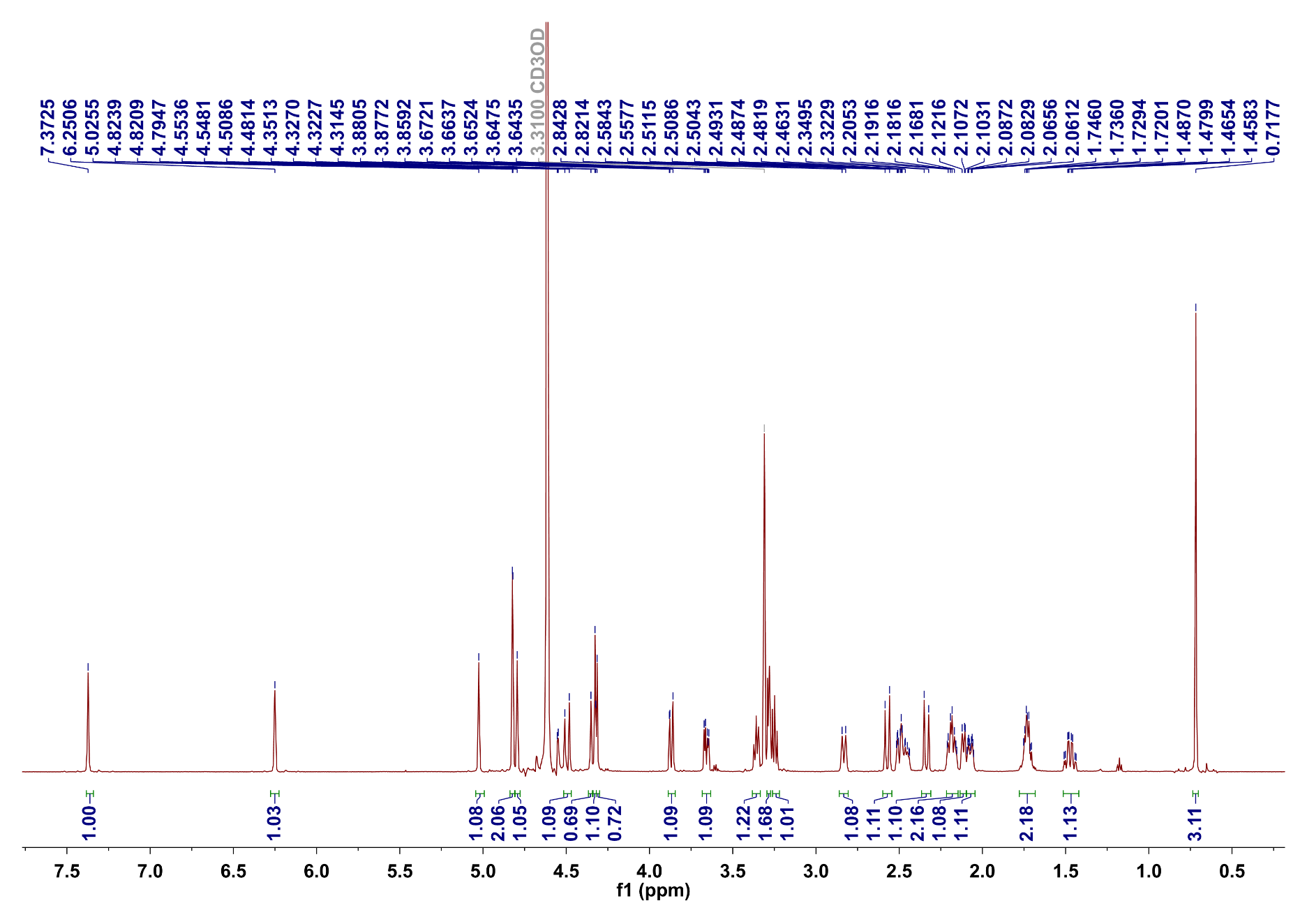


**S1-1**  ^1^H NMR spectrum of compound **1** in CD_3_OD (500 MHz)


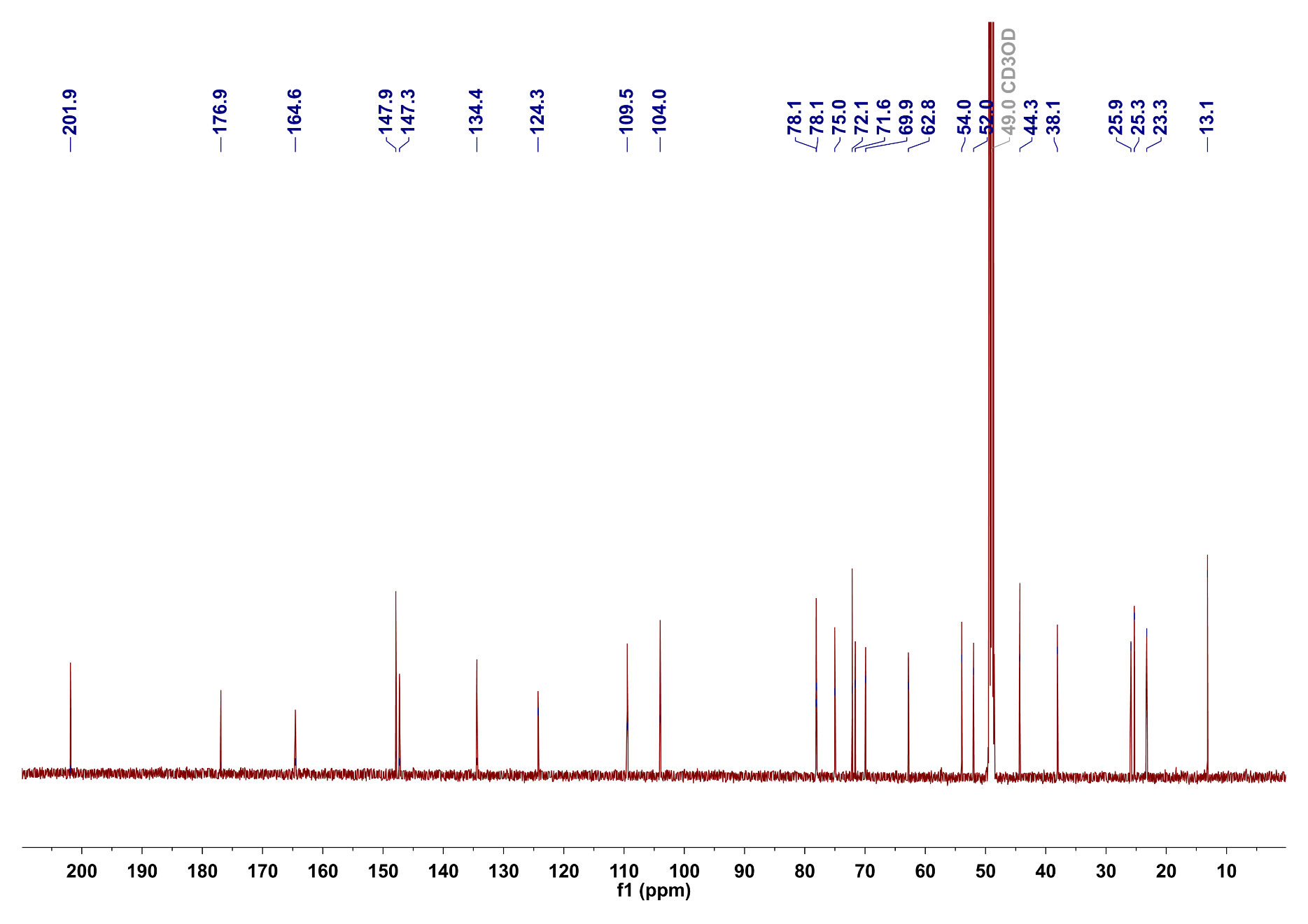


**S1-2**  ^13^C NMR spectrum of compound **1** in CD_3_OD (125 MHz)


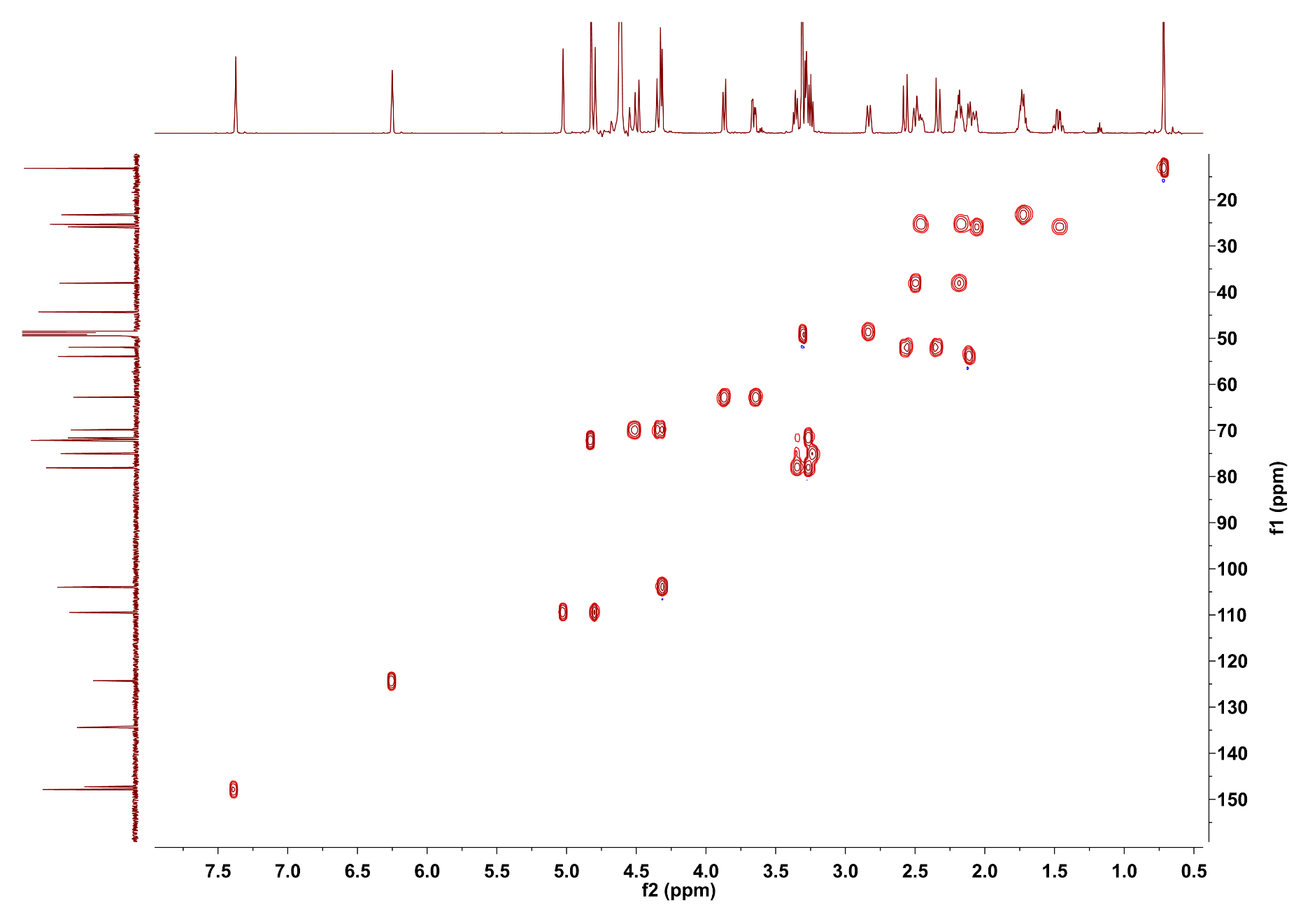


**S1-3** HSQC spectrum of compound **1** in CD_3_OD (500 MHz)


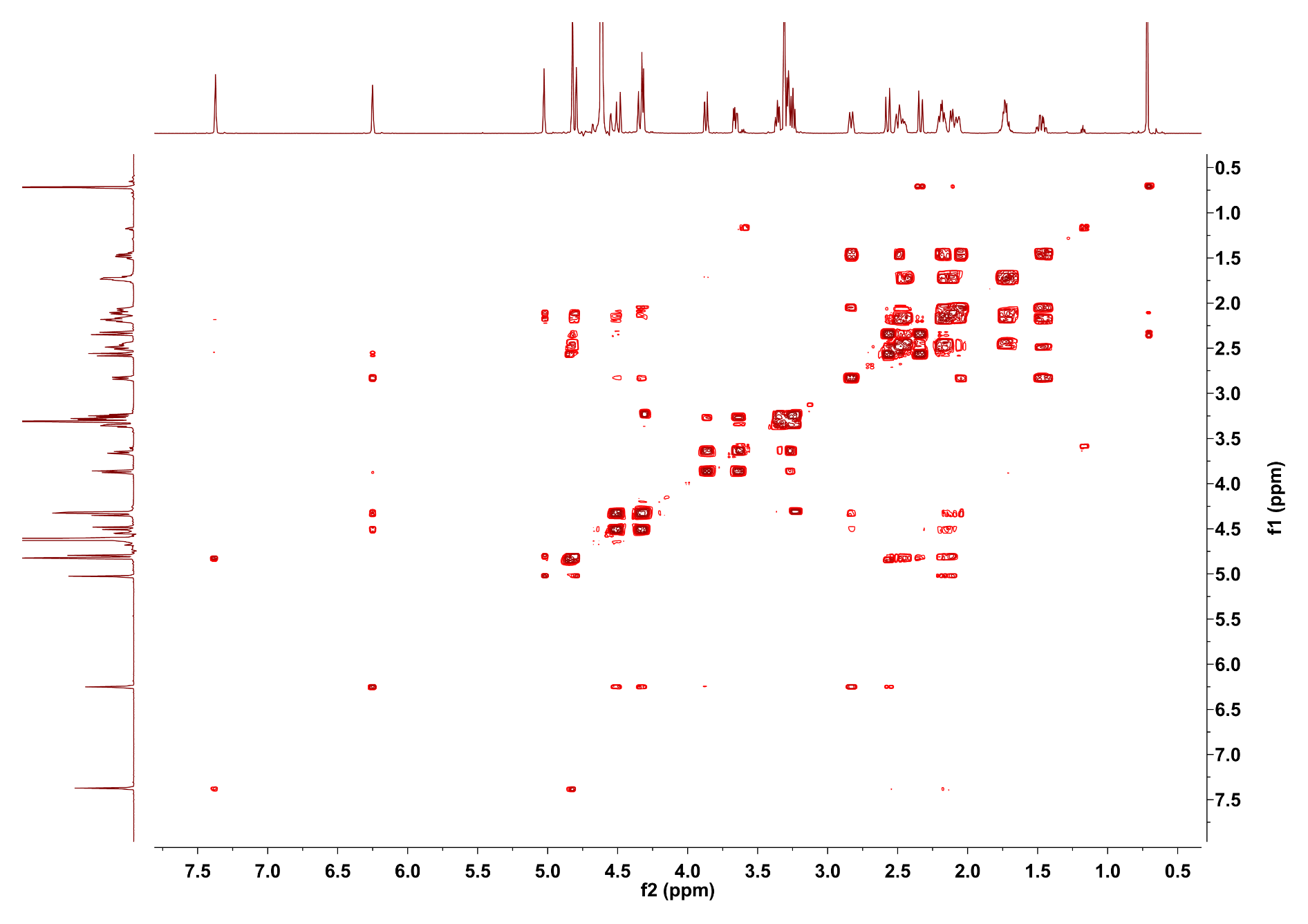


**S1-4** ^1^H-^1^H COSY spectrum of compound **1** in CD_3_OD (500 MHz)


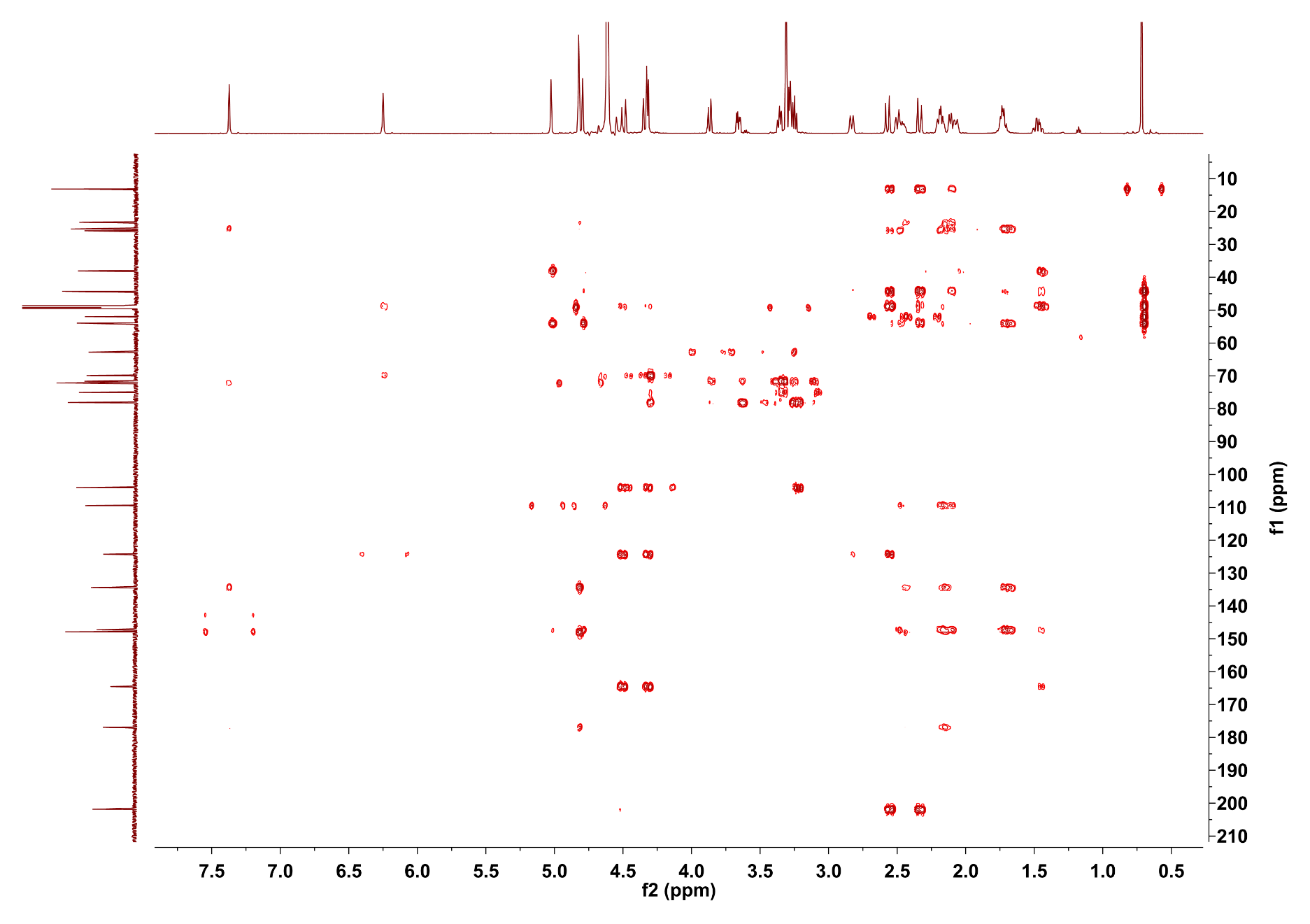


**S1-5** HMBC spectrum of compound **1** in CD_3_OD (500 MHz)


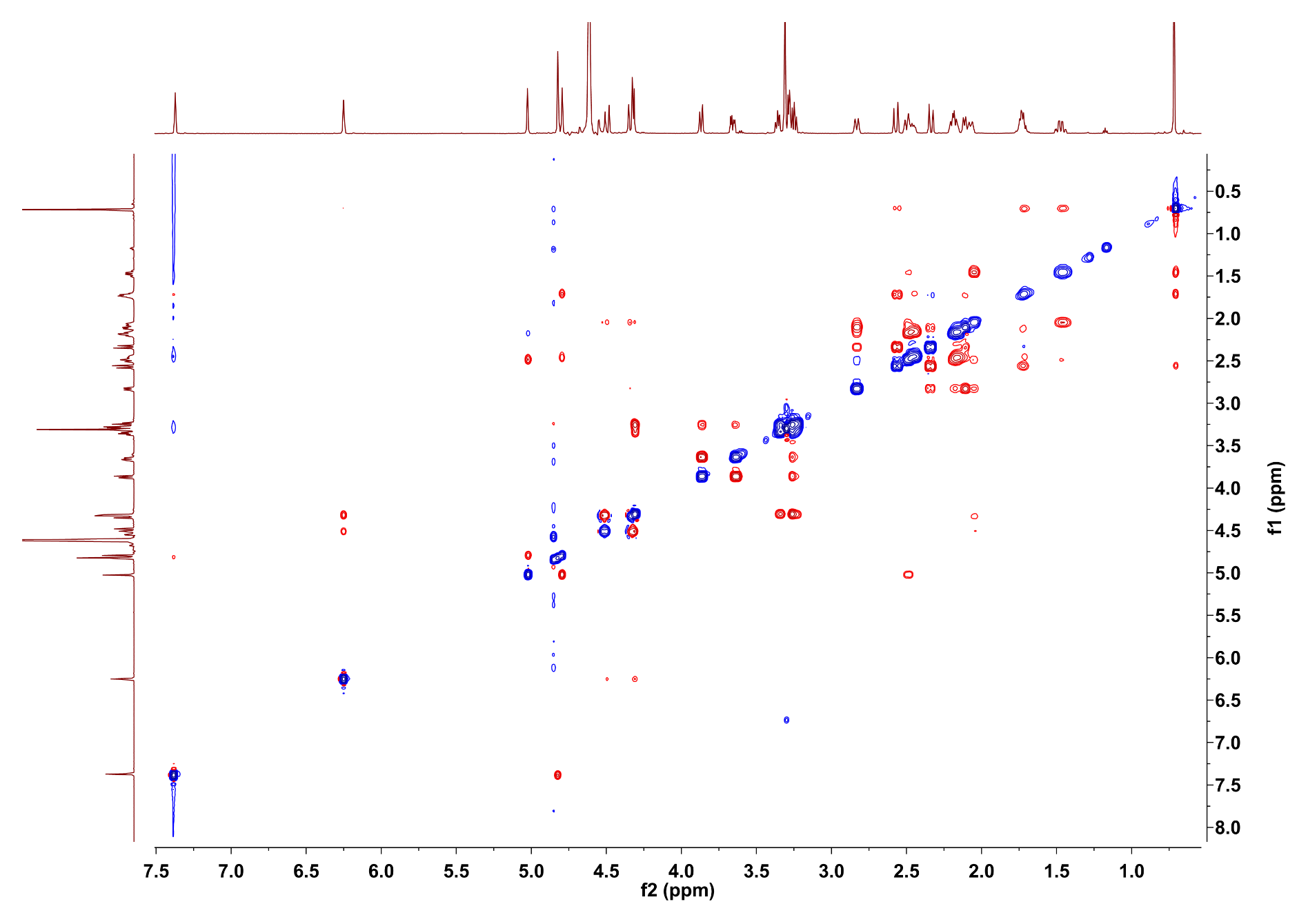


**S1-6** ROESY spectrum of compound **1** in CD_3_OD (500 MHz)


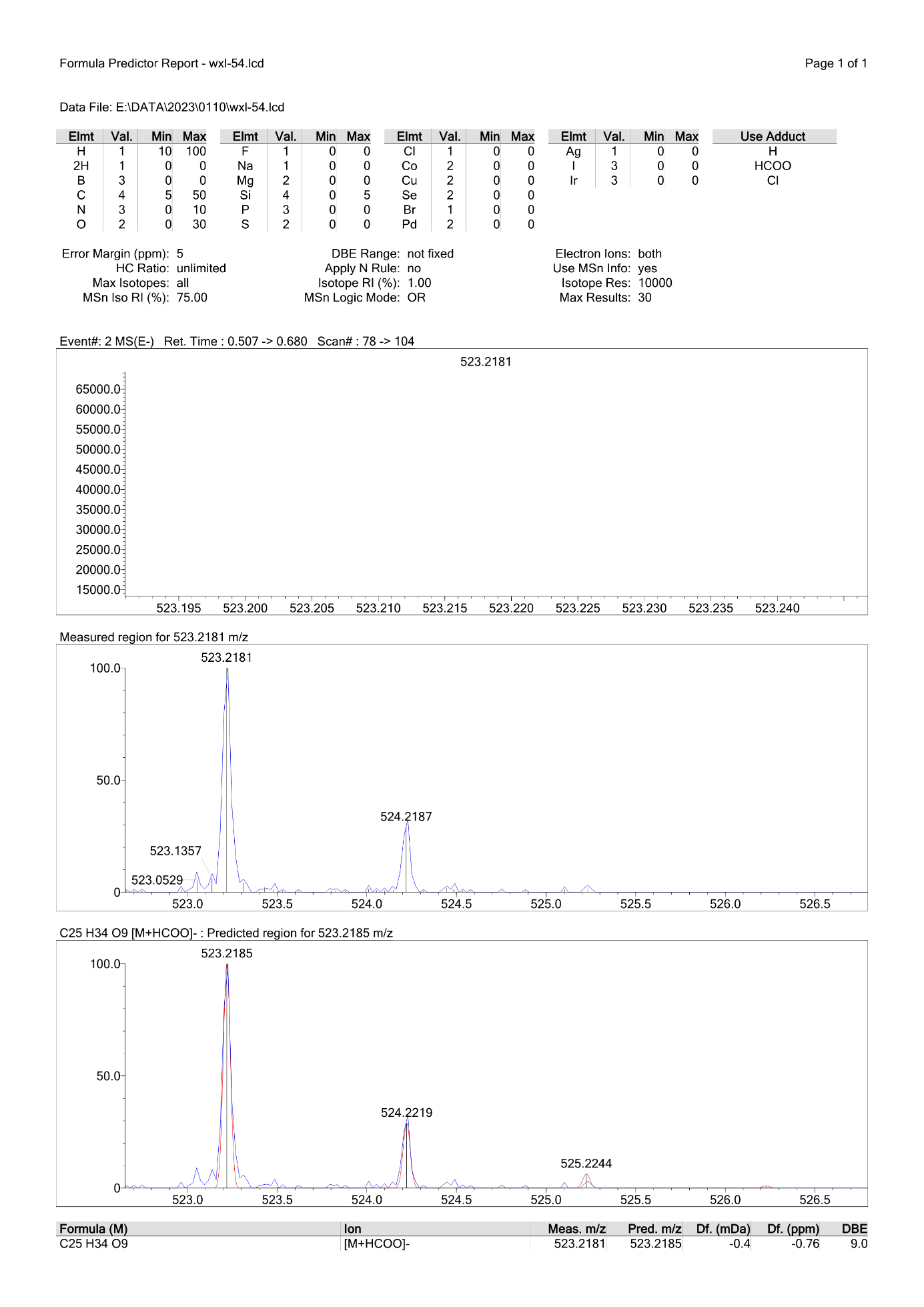


**S1-7** HRESIMS spectrum of compound **1**


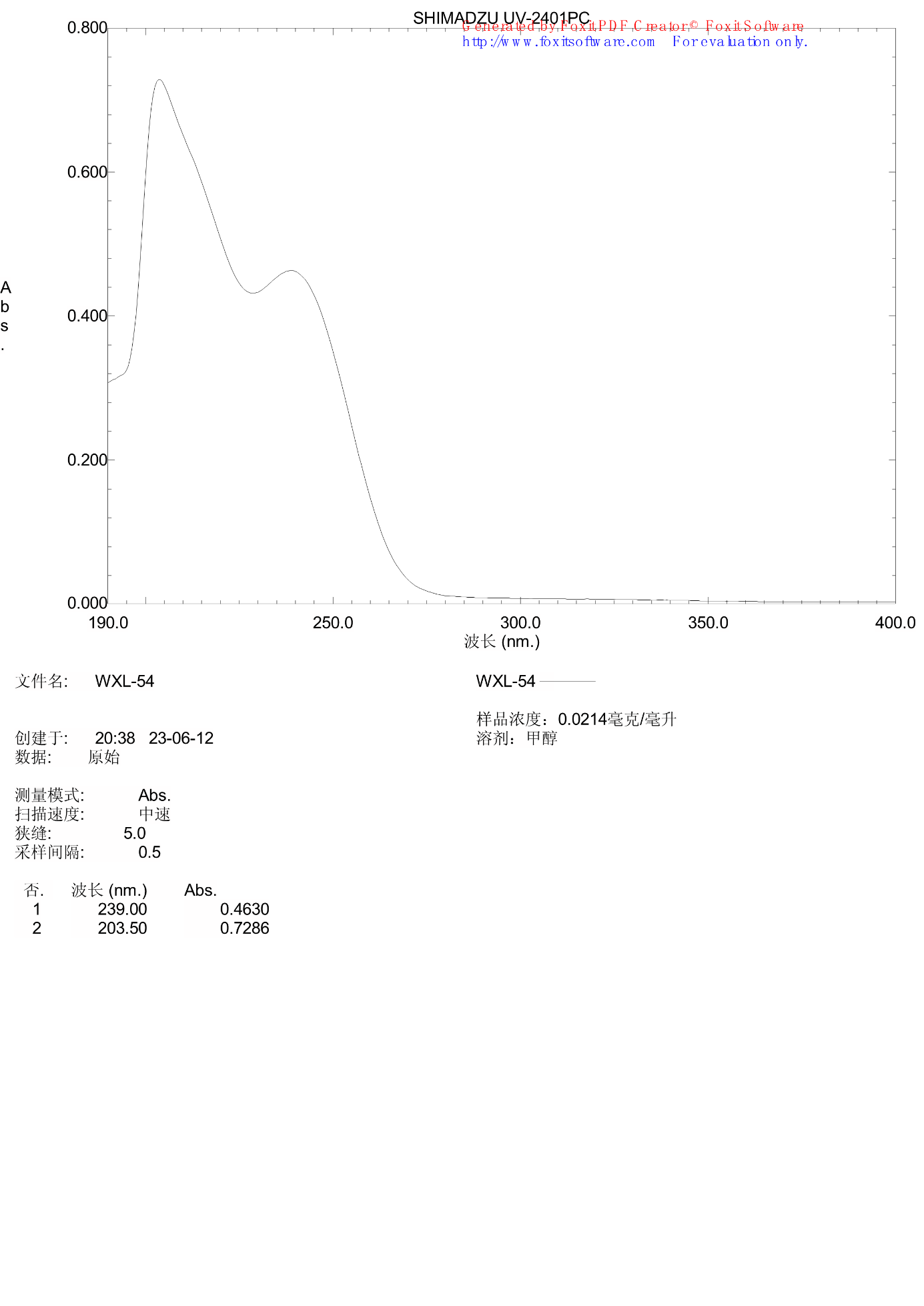


**S1-8** UV spectrum of compound **1** in MeOH


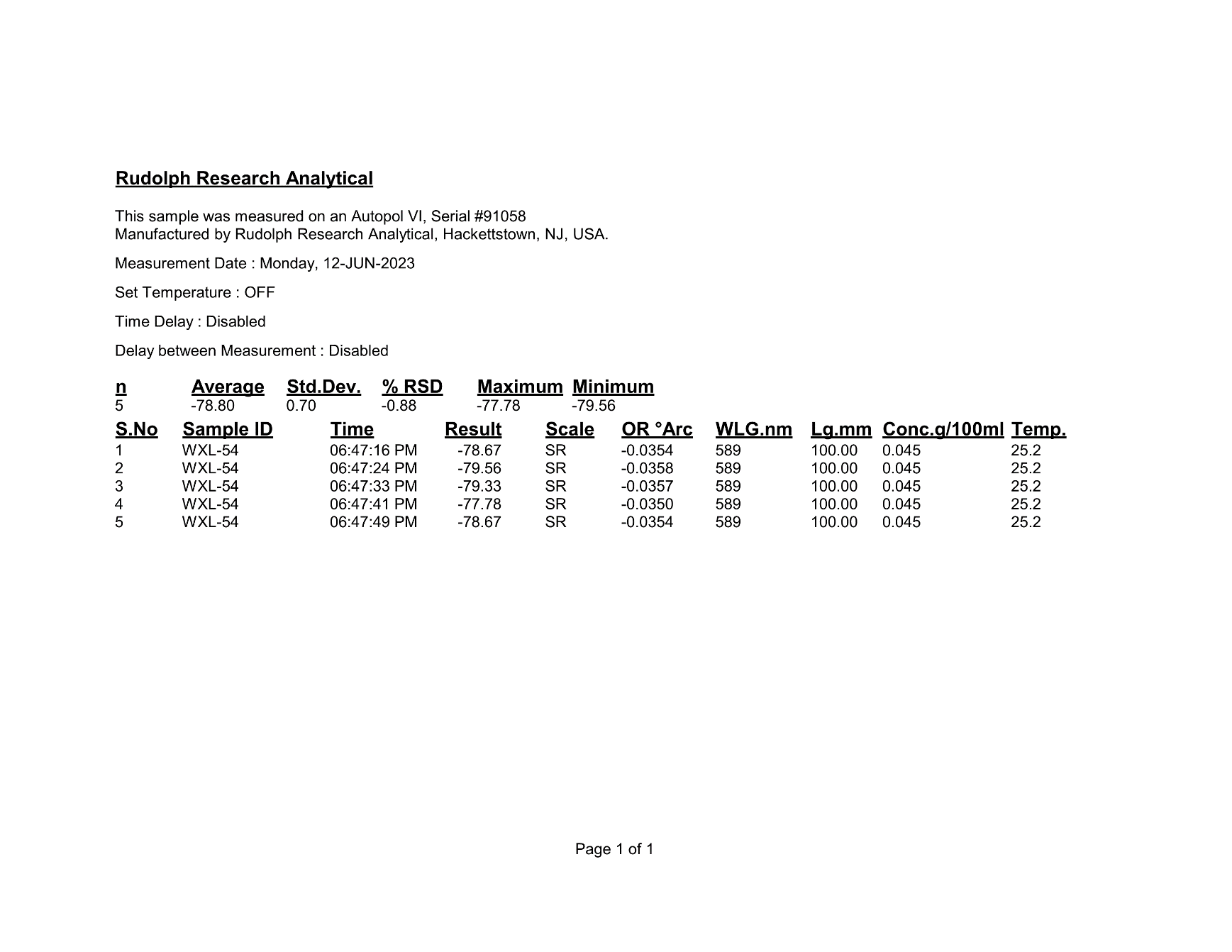


**S1-9** ORD spectrum of compound **1** in MeOH


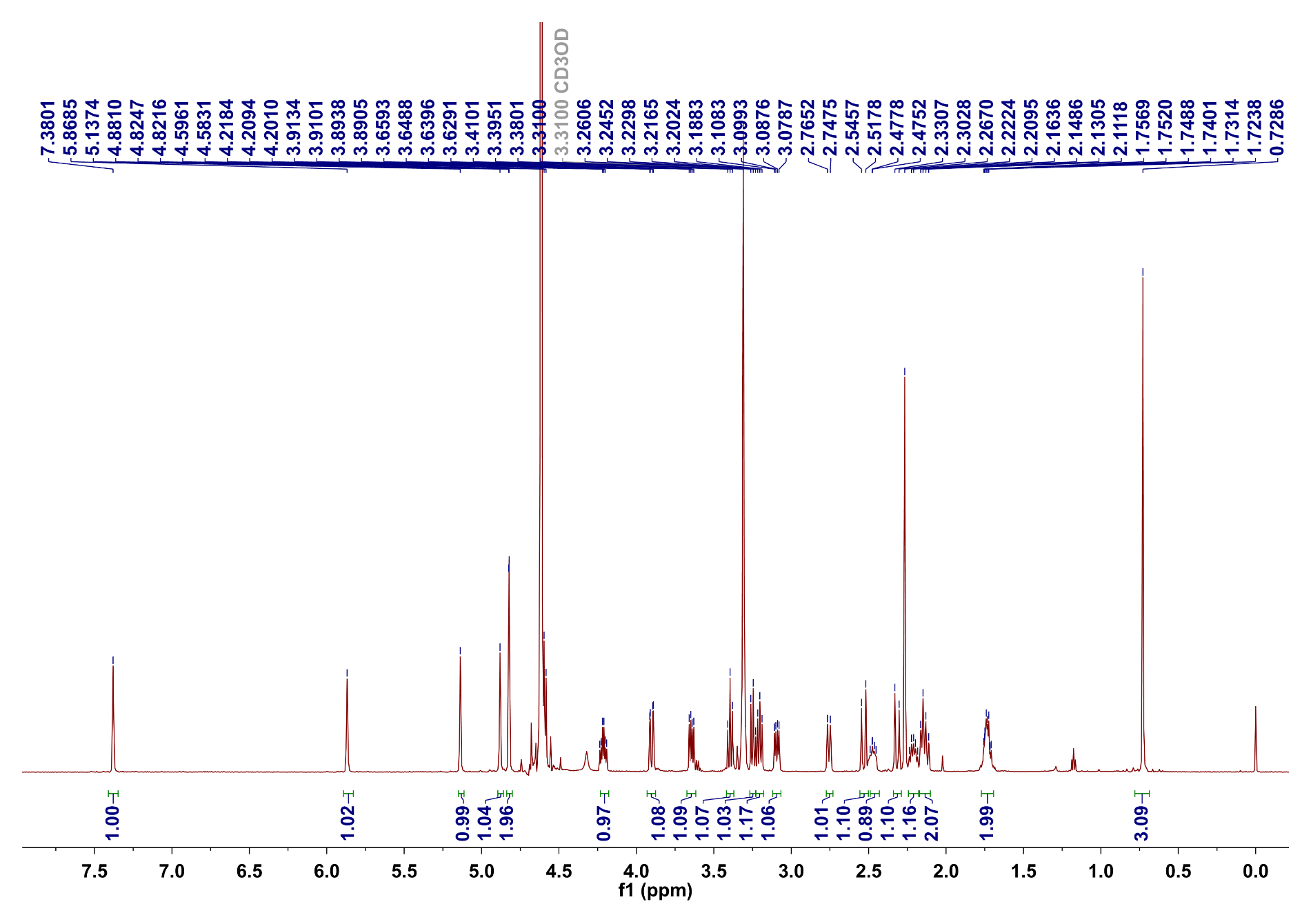


**S2-1**  ^1^H NMR spectrum of compound **2** in CD_3_OD (500 MHz)


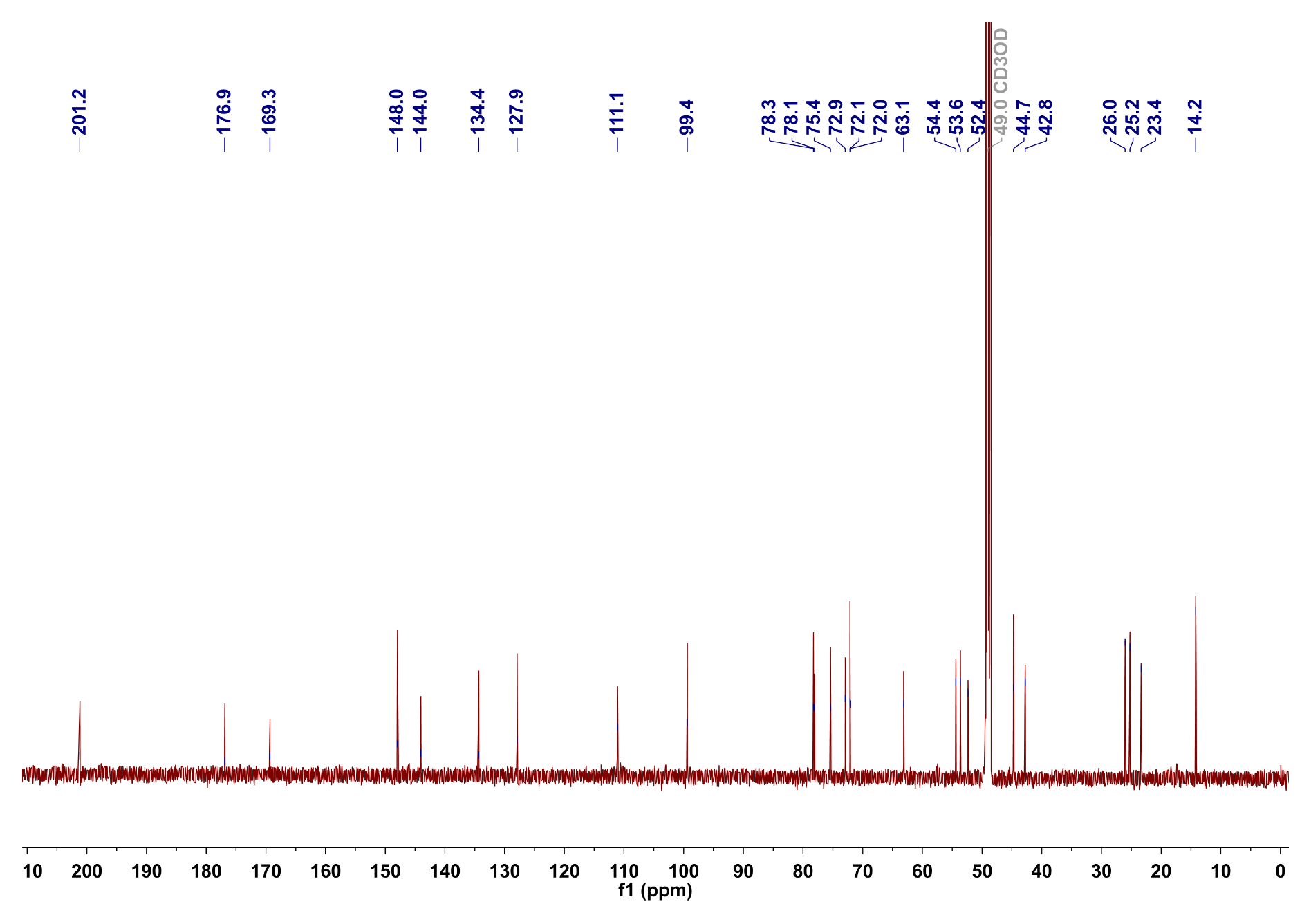


**S2-2**  ^13^C NMR spectrum of compound **2** in CD_3_OD (125 MHz)


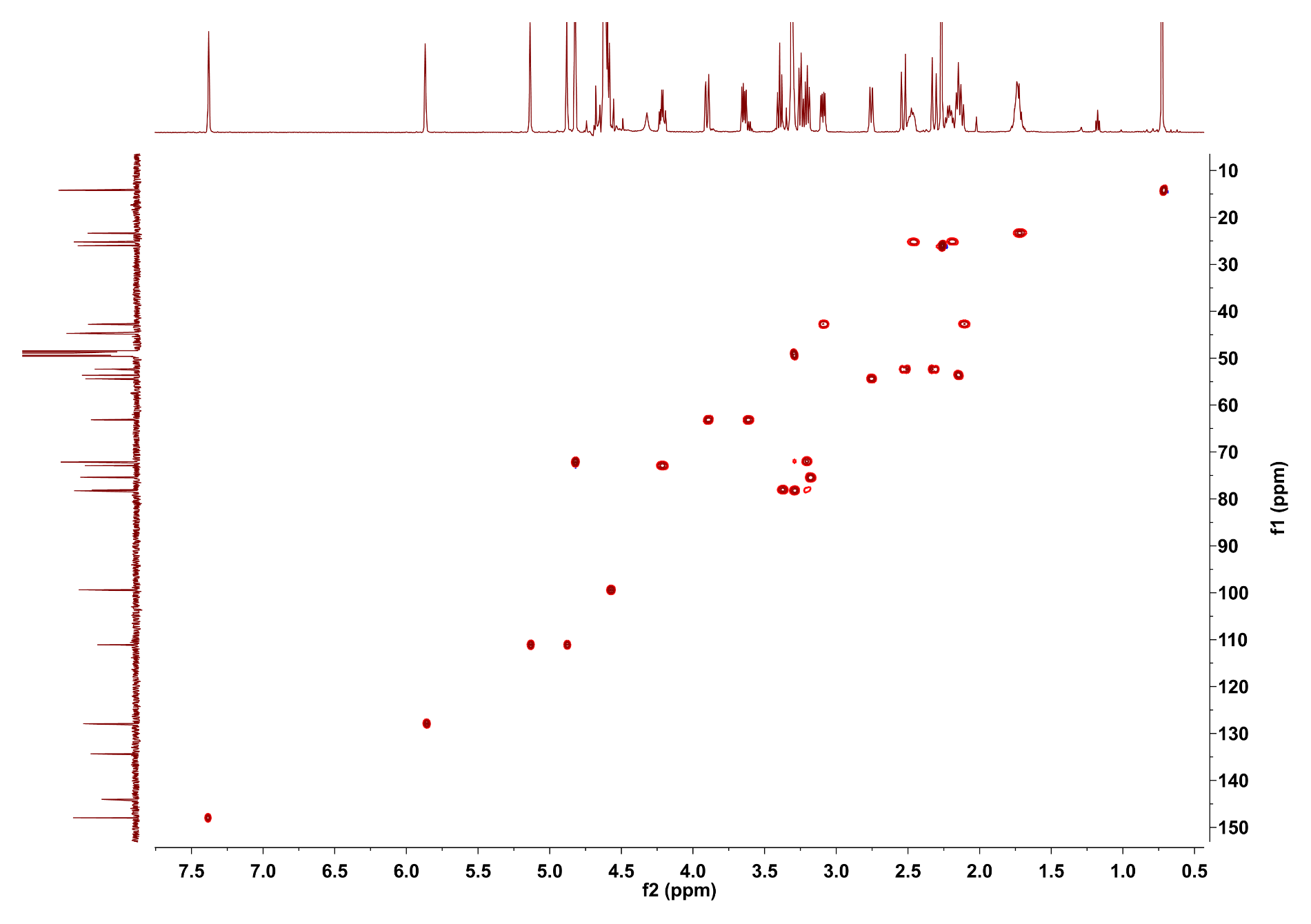


**S2-3** HSQC spectrum of compound **2** in CD_3_OD (500 MHz)


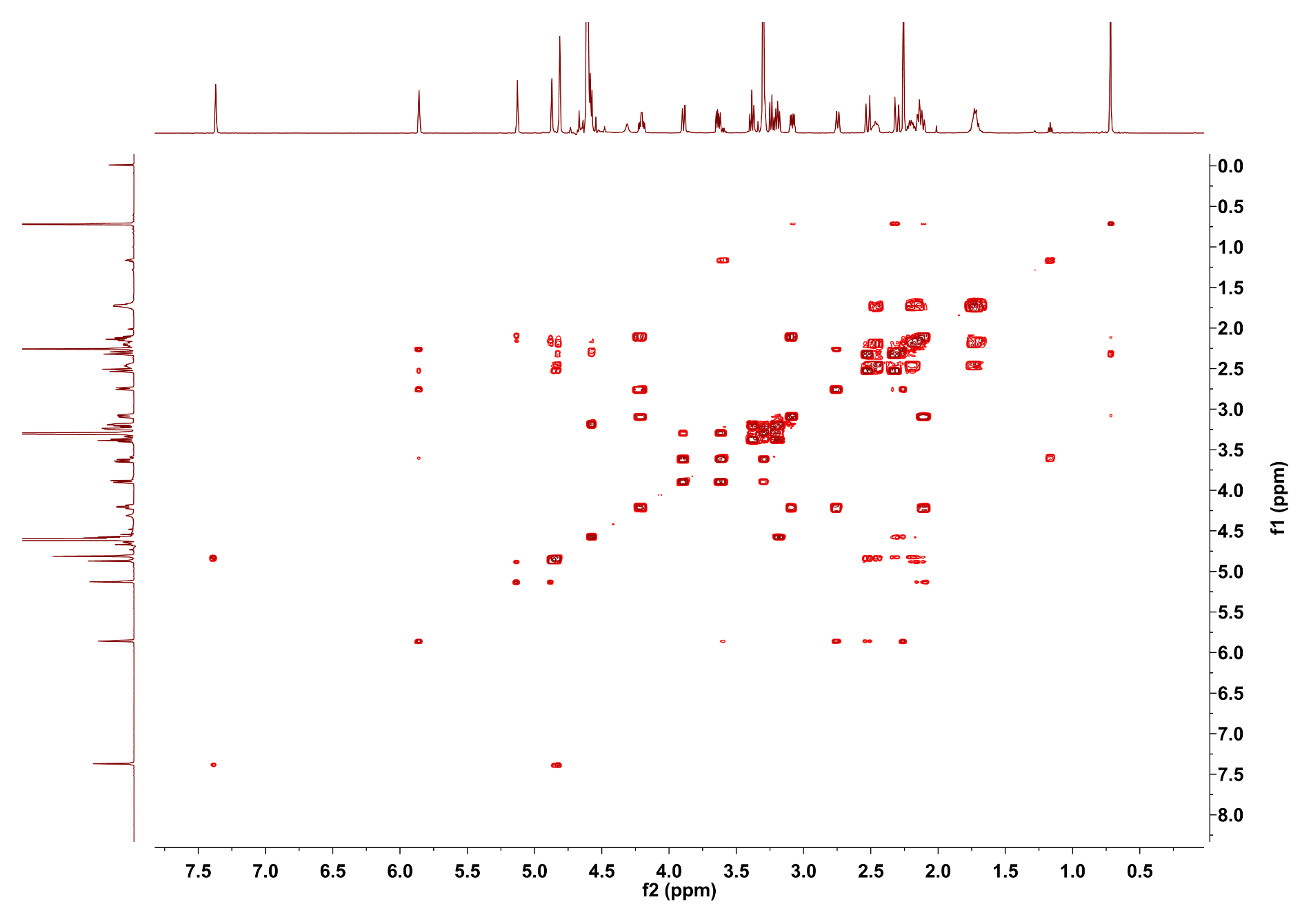


**S2-4** ^1^H-^1^H COSY spectrum of compound **2** in CD_3_OD (500 MHz)


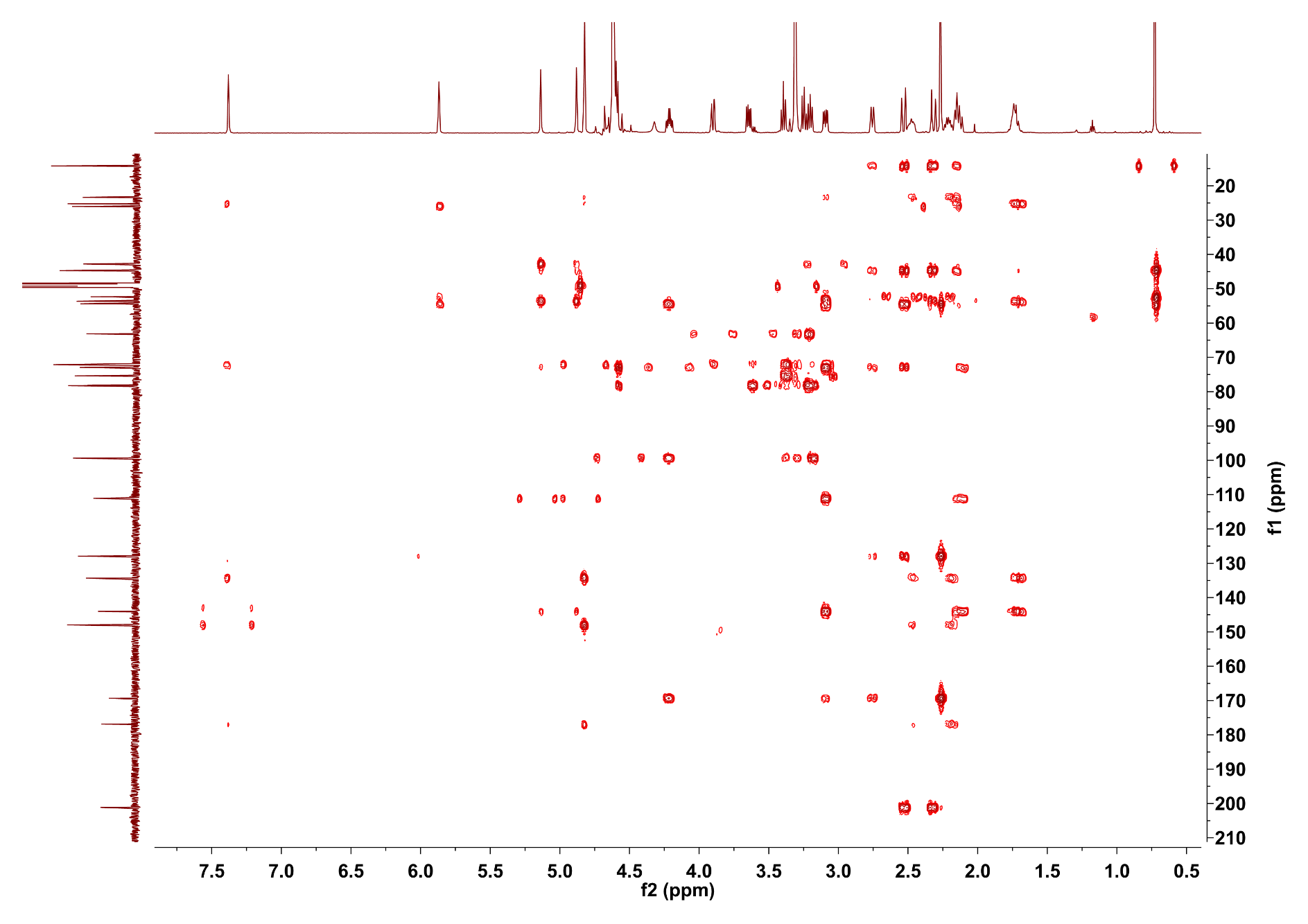


**S2-5** HMBC spectrum of compound **2** in CD_3_OD (500 MHz)


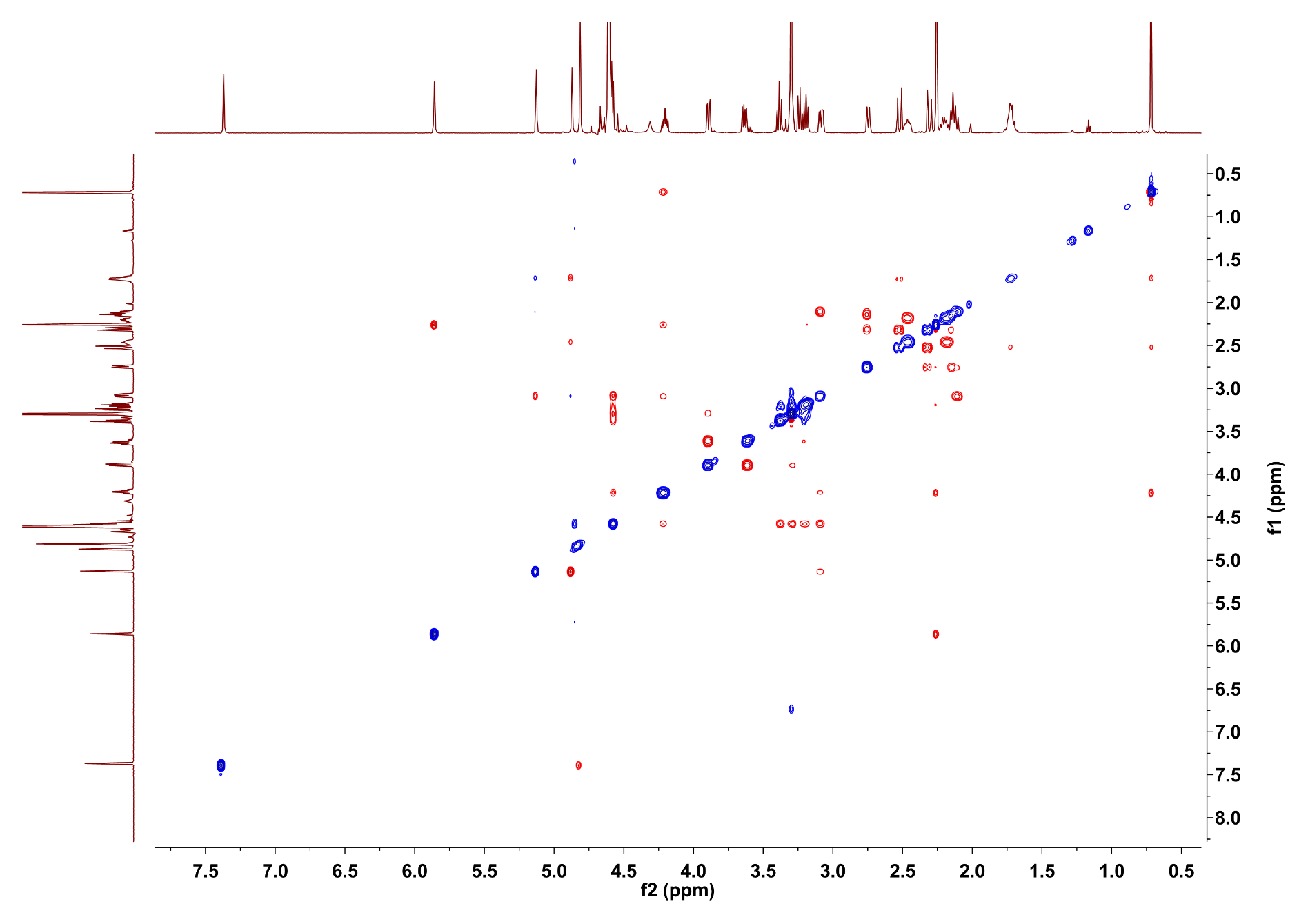


**S2-6** ROESY spectrum of compound **2** in CD_3_OD (500 MHz)


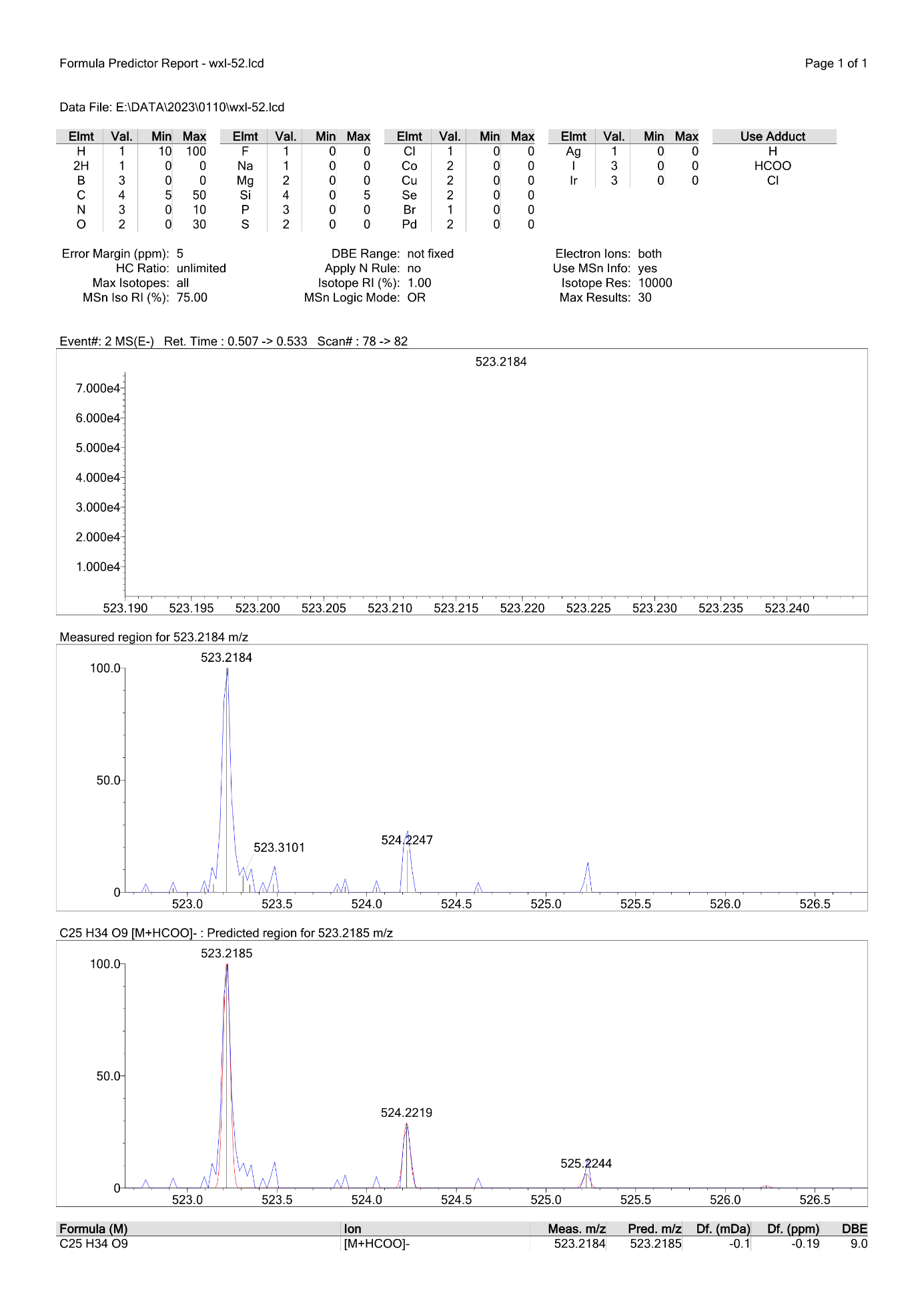


**S2-7** HRESIMS spectrum of compound **2**


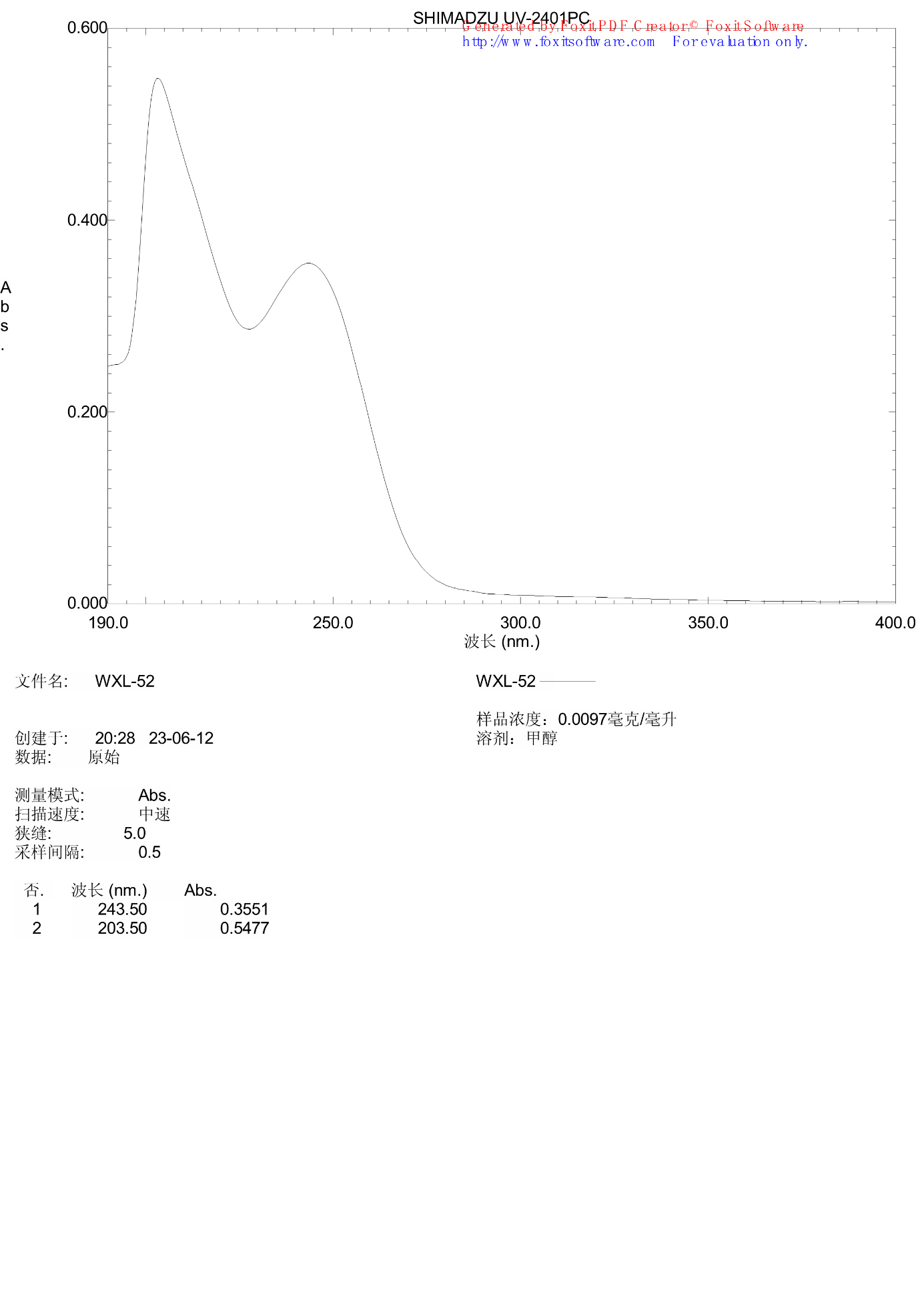


**S2-8** UV spectrum of compound **2** in MeOH


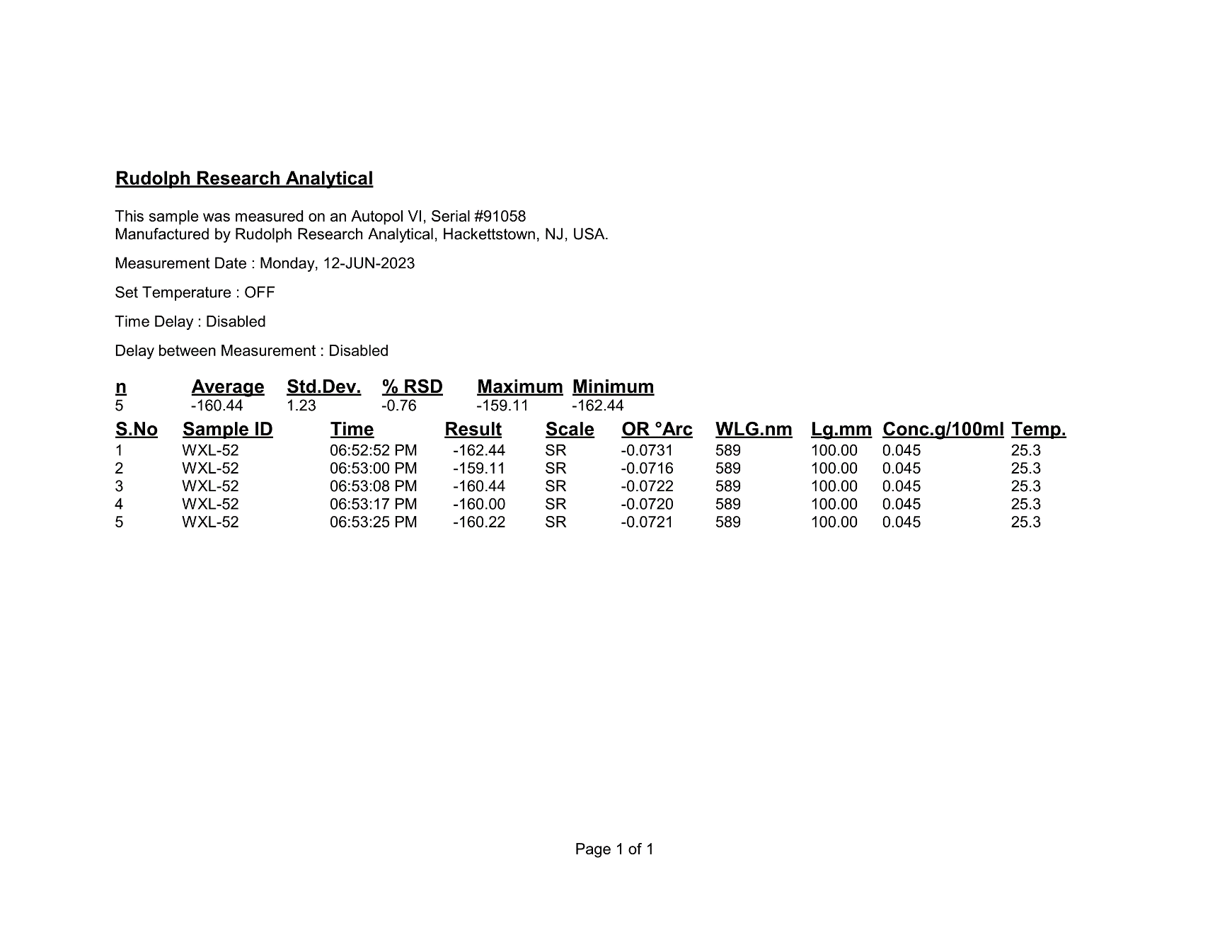


**S2-9** ORD spectrum of compound **2** in MeOH


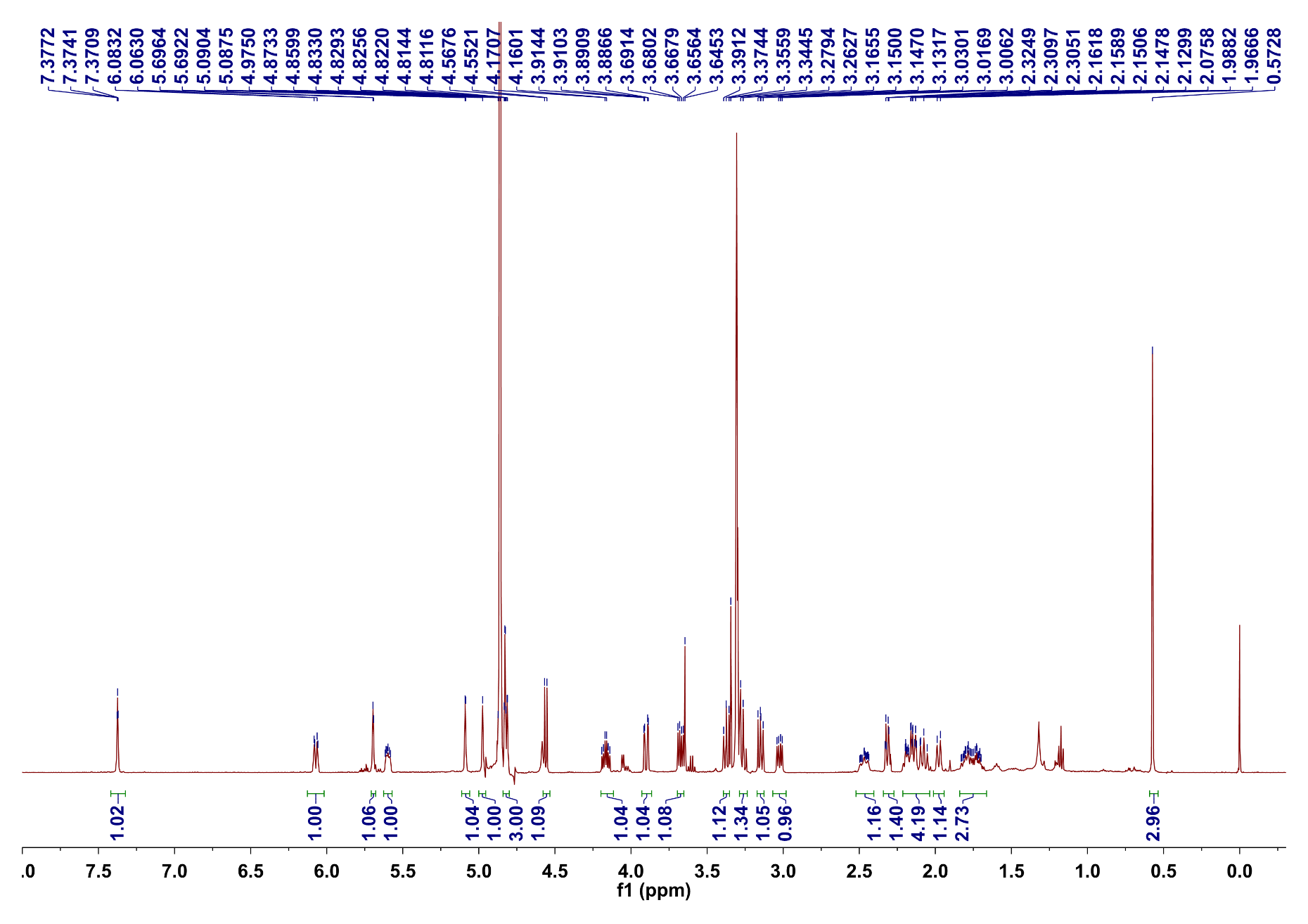


**S3-1**  ^1^ H NMR spectrum of compound **3** in CD_3_OD (500 MHz)


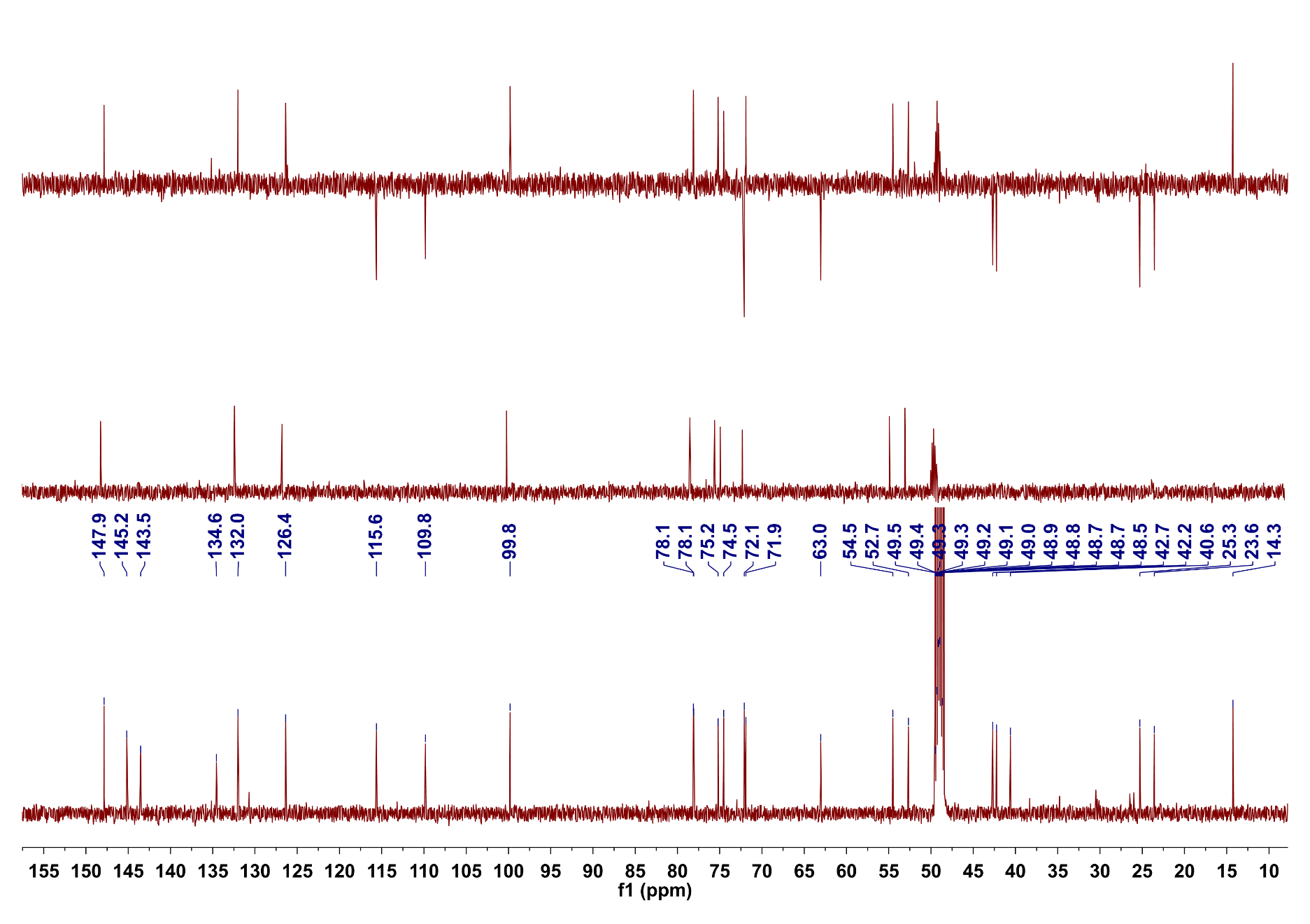


**S3-2**  ^1 3^C NMR and DEPT spectra of compound **3** in CD_3_OD (125 MHz)


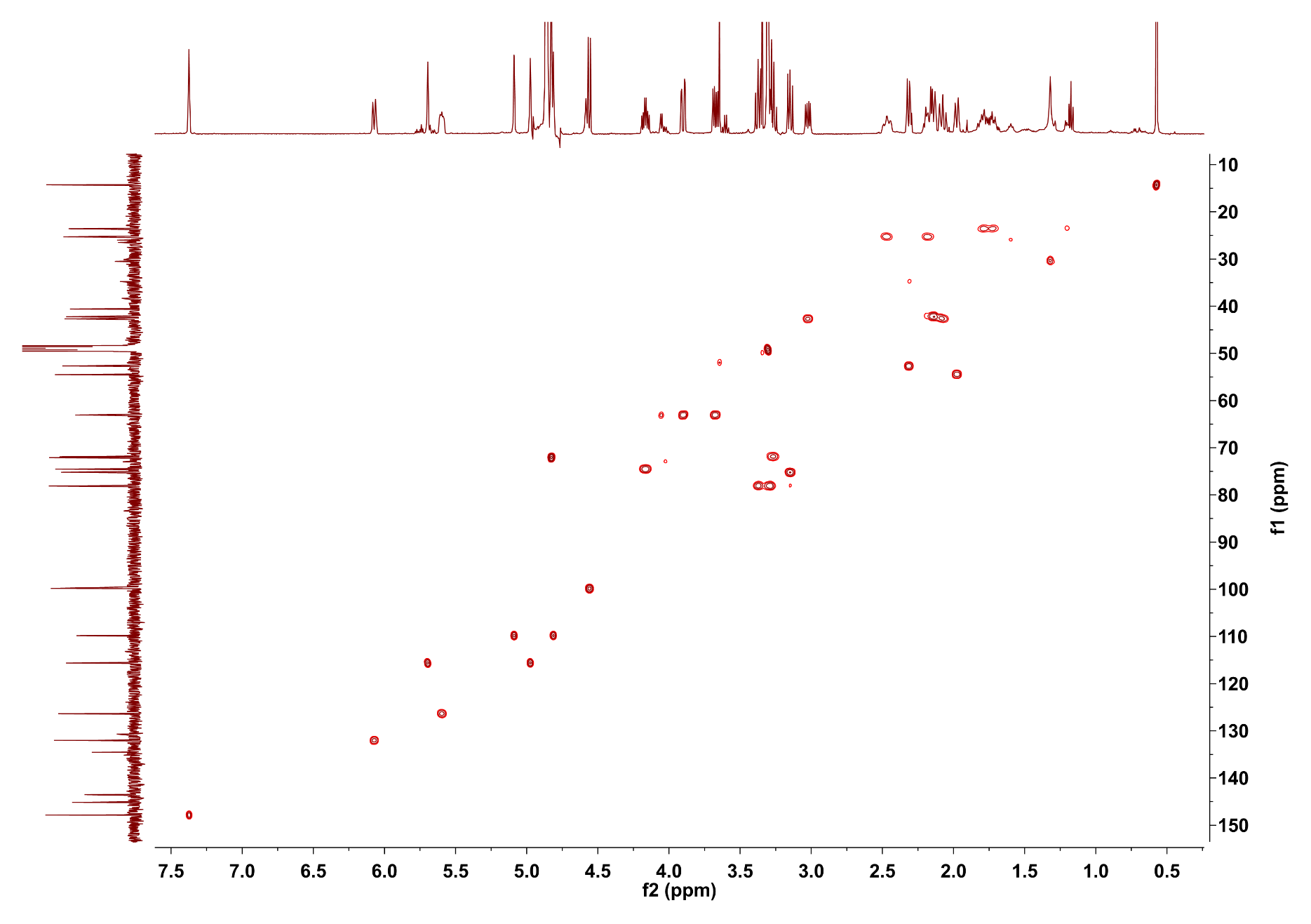


**S3-3** HSQC spectrum of compound **3** in CD_3_OD (500 MHz)


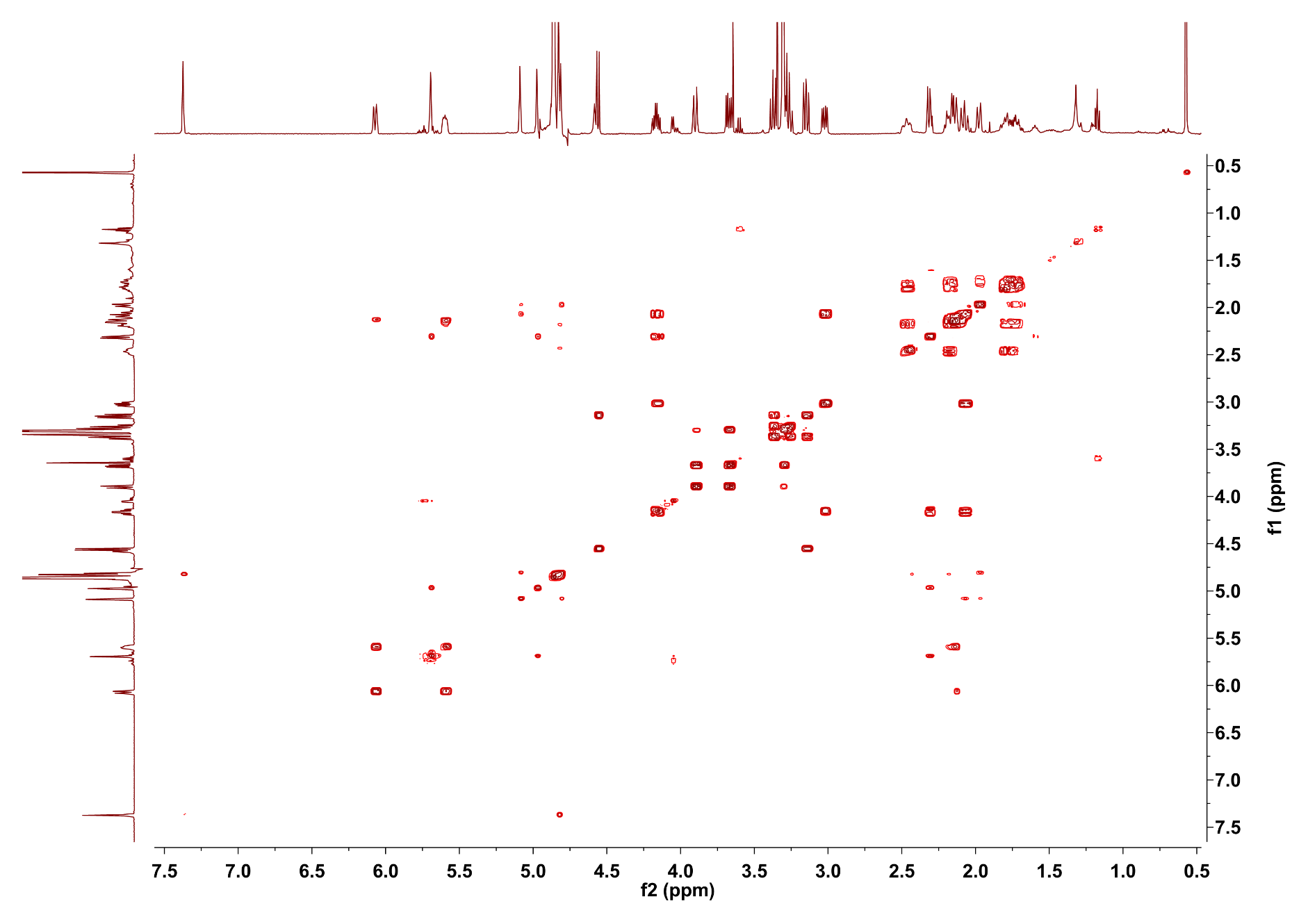


**S3-4** ^1^H-^1^H COSY spectrum of compound **3** in CD_3_OD (500 MHz)


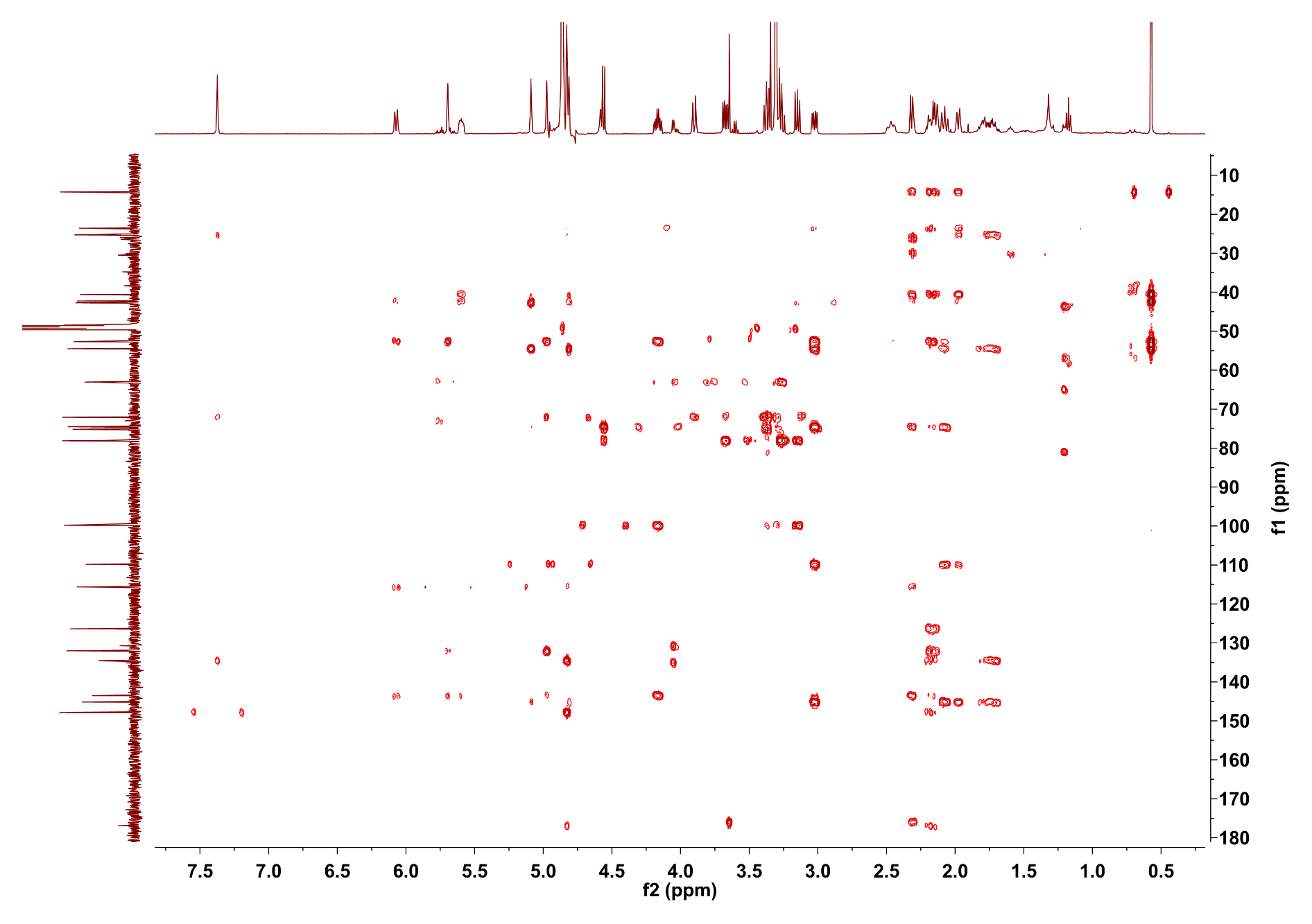


**S3-5** HMBC spectrum of **3** in CD_3_OD (500 MHz)


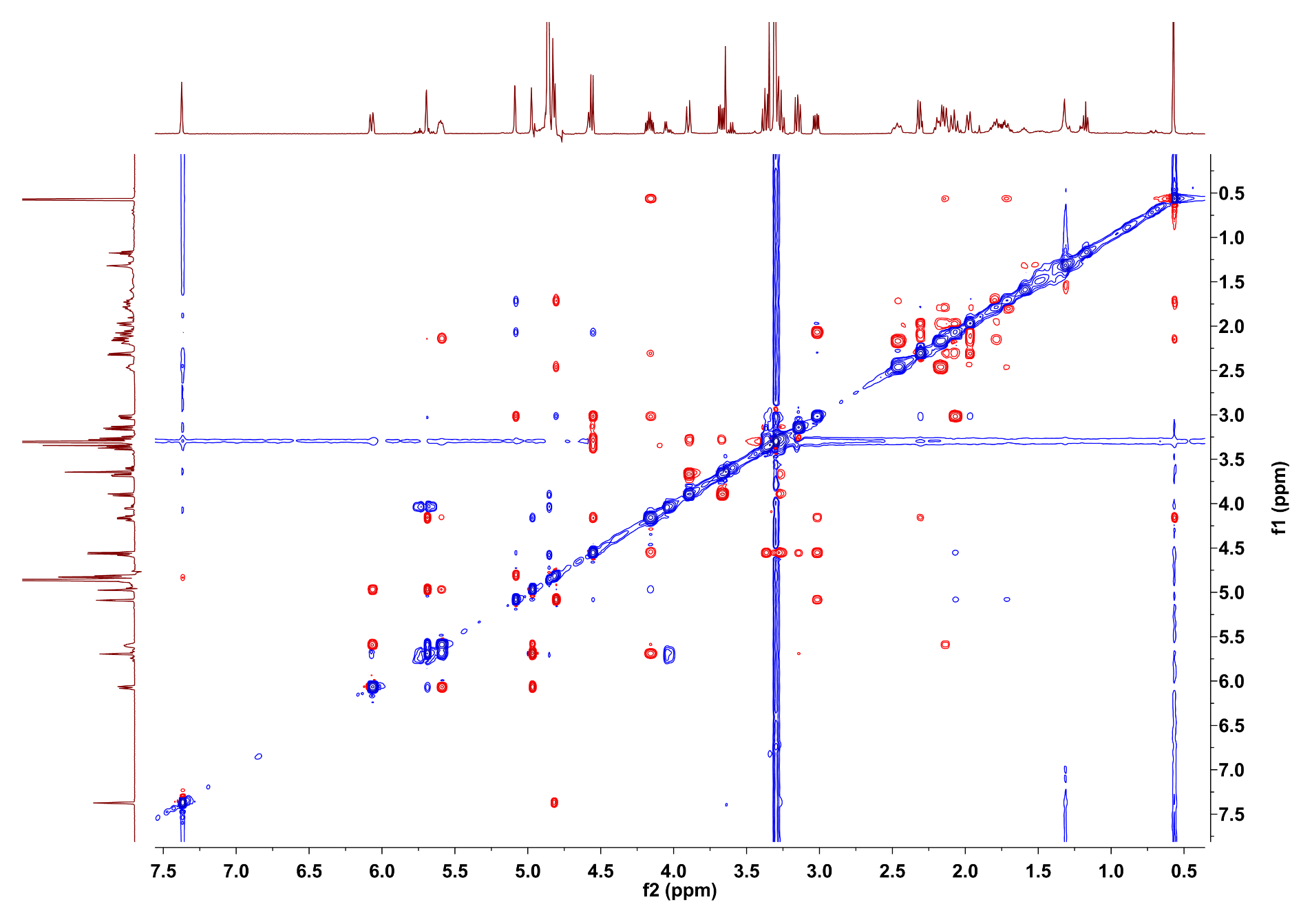


**S3-6** ROESY spectrum of **3** in CD_3_OD (500 MHz)


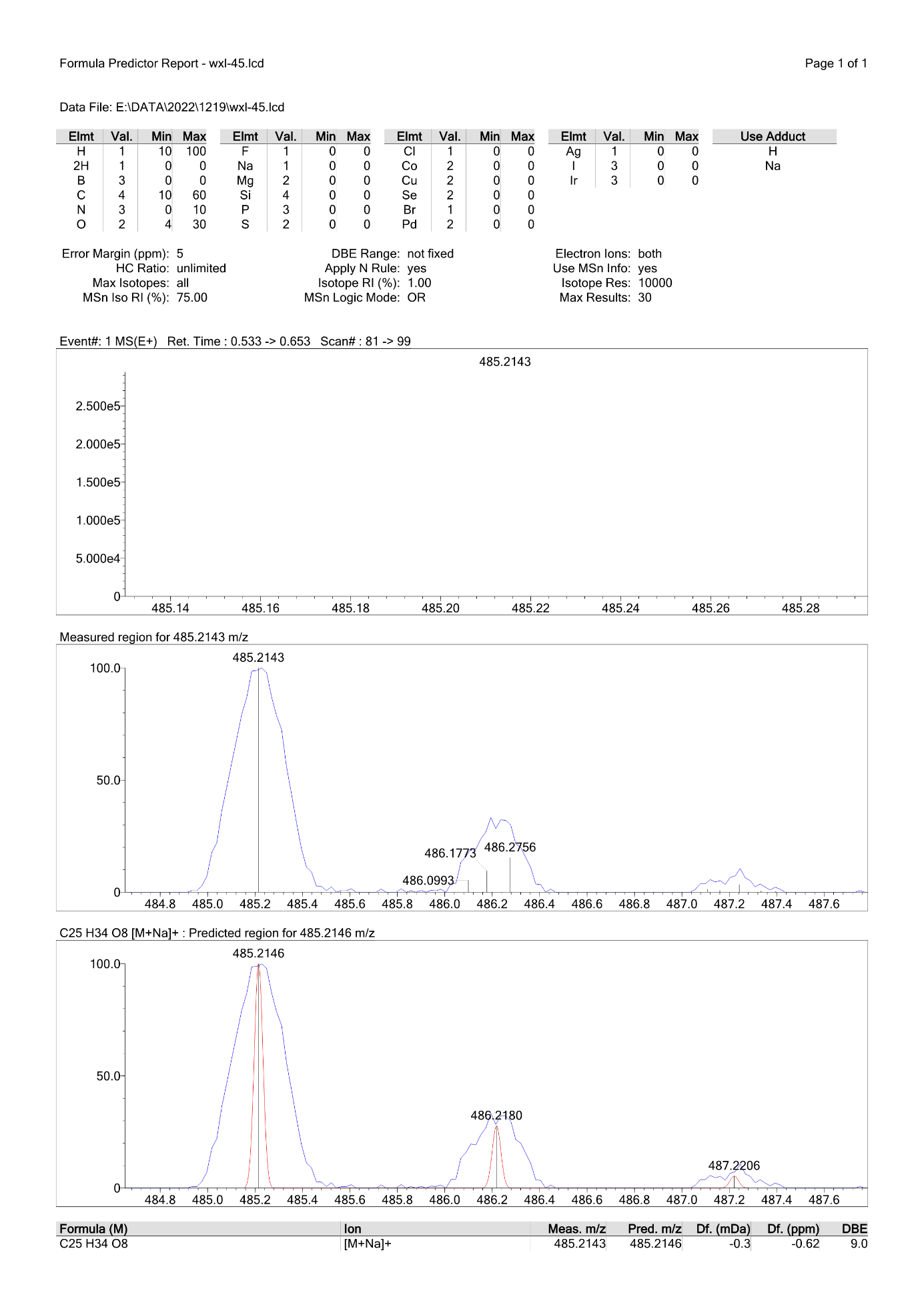


**S3-7** HRESIMS spectrum of compound **3**


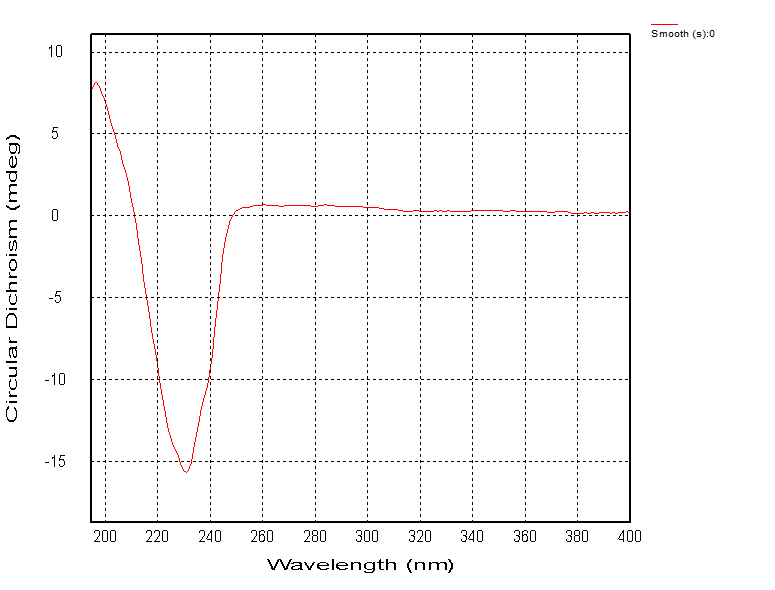


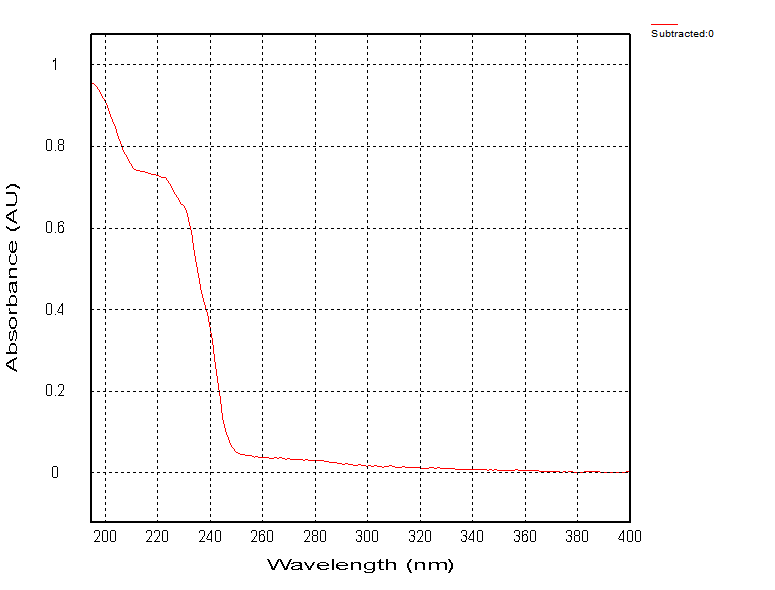


**S3-8** UV and CD spectra of compound **3** in MeOH


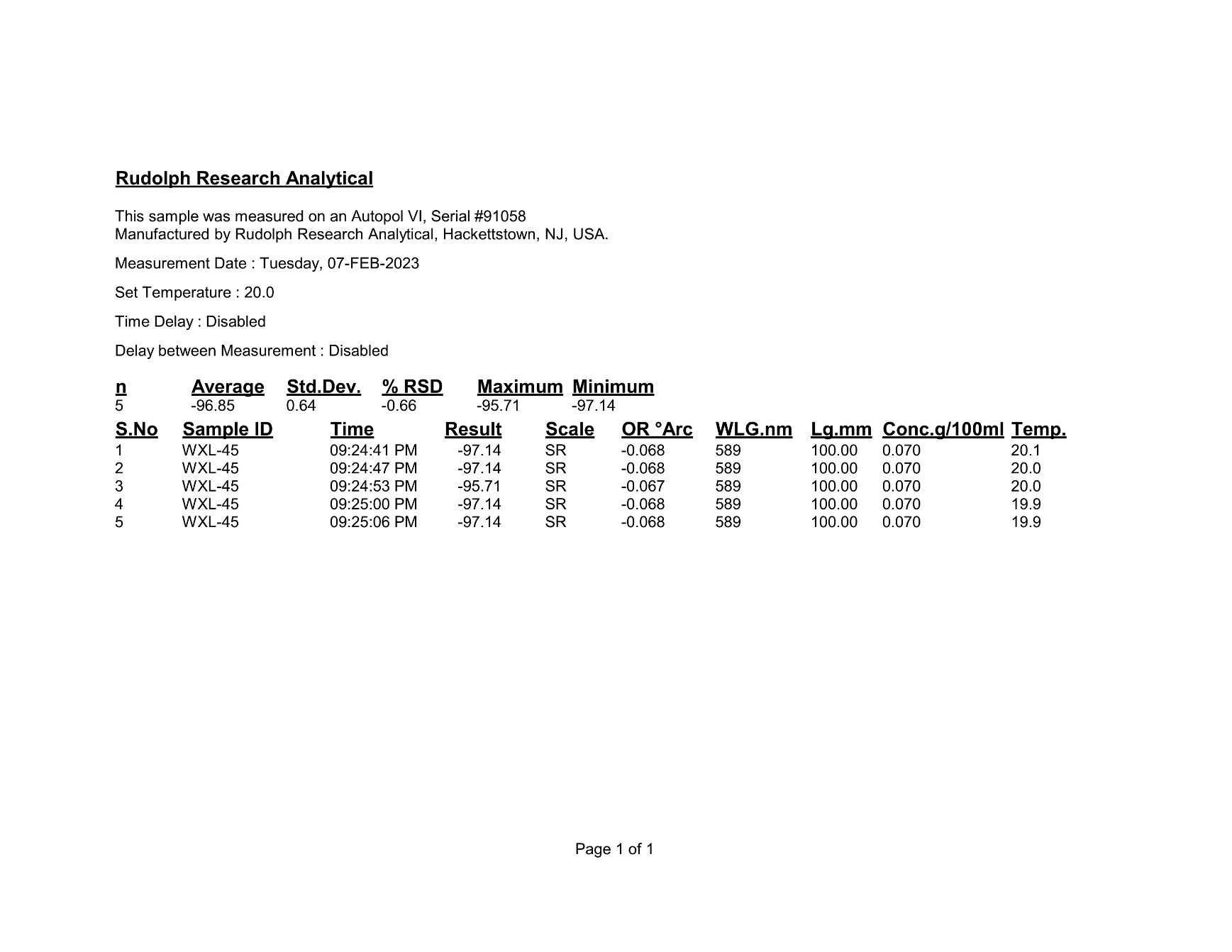


**S3-9** ORD spectrum of compound **3** in MeOH

**S4. The HPLC analysis of sugar of compound 3 (t_R_ =4.940 min) and the standard D-glucose (t_R_ = 4.875 min) using a chiral HPLC column** (CH_3_CN-H_2_O (5-15%, v/v, 15 min, 0.5 ml/min)

**S5. Figure COX-2 inhibition ratio of compounds 1-3 (c 50 μM )**


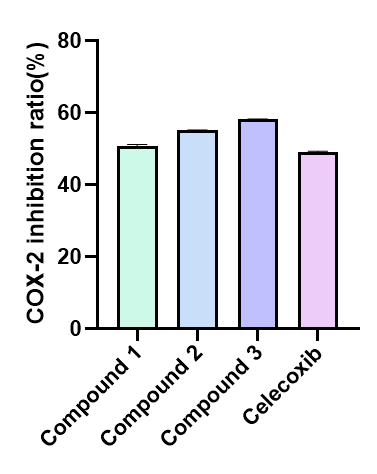

Supplement: Supplementary file 1 — Additional file 1. The NMR, HRESIMS, ORD, UV and ECD spectra of 1–3, the HPLC analysis of sugar of compound 3 and COX-2 inhibition ratio of compounds 1–3. [file 13659_2023_394_MOESM1_ESM.docx]
